# Structural insights into the assembly and activation of the IL‐27 signaling complex

**DOI:** 10.15252/embr.202255450

**Published:** 2022-08-03

**Authors:** Yibo Jin, Paul K Fyfe, Scott Gardner, Stephan Wilmes, Doryen Bubeck, Ignacio Moraga

**Affiliations:** ^1^ Department of Life Sciences, Sir Ernst Chain Building Imperial College London London UK; ^2^ Division of Cell Signaling and Immunology, School of Life Sciences University of Dundee Dundee UK

**Keywords:** cytokine, IL‐27, cryo electron microscopy, GP130, Immunology, Signal Transduction, Structural Biology

## Abstract

Interleukin 27 (IL‐27) is a heterodimeric cytokine that elicits potent immunosuppressive responses. Comprised of EBI3 and p28 subunits, IL‐27 binds GP130 and IL‐27Rα receptor chains to activate the JAK/STAT signaling cascade. However, how these receptors recognize IL‐27 and form a complex capable of phosphorylating JAK proteins remains unclear. Here, we used cryo electron microscopy (cryoEM) and AlphaFold modeling to solve the structure of the IL‐27 receptor recognition complex. Our data show how IL‐27 serves as a bridge connecting IL‐27Rα (domains 1–2) with GP130 (domains 1–3) to initiate signaling. While both receptors contact the p28 component of the heterodimeric cytokine, EBI3 stabilizes the complex by binding a positively charged surface of IL‐27Rα and Domain 1 of GP130. We find that assembly of the IL‐27 receptor recognition complex is distinct from both IL‐12 and IL‐6 cytokine families and provides a mechanistic blueprint for tuning IL‐27 pleiotropic actions.

## Graphical abstract

Interleukin 27 is a heterodimeric cytokine that elicits potent immuno‐suppressive responses. The structure of the IL‐27 signalling complex provides a mechanistic blueprint to fine‐tune IL‐27 immuno‐modulatory activities.

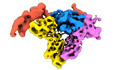

## Introduction

IL‐27 is an immunosuppressive cytokine involved in resolving T cell‐mediated inflammation. IL‐27 inhibits Th‐17 responses (Stumhofer *et al*, 2006; Yang *et al*, 2008; Diveu *et al*, 2009) and induces differentiation of T regulatory cells (Hall *et al*, 2012). T cell stimulation by IL‐27 promotes secretion of the anti‐inflammatory cytokine, IL‐10, further contributing to a reduction in the inflammatory response (Stumhofer *et al*, 2007). Together, these properties make IL‐27 an attractive drug target to treat T cell‐mediated inflammatory disease. IL‐27 also contributes to immune exhaustion by regulating expression of co‐inhibitory receptors (Chihara *et al*, 2018; DeLong *et al*, 2019). Elevated levels of IL‐27 gene signatures are found in cancer and are associated with poor prognoses (Jia *et al*, 2016). Understanding how IL‐27 engages its cellular receptors, GP130 and IL‐27Rα, to form a signaling complex will provide fundamental insight into immune regulation, which could facilitate the development of new therapeutics that target IL‐27 responses.

IL‐27 is a heterodimeric cytokine composed of two gene products, Epstein–Barr virus‐induced gene 3 (EBI3) and IL‐27p28 (p28; Vignali & Kuchroo, 2012). Grouped within the IL‐12 family of heterocytokines, p28 and EBI3 share sequence homology with two components of IL‐12: p35 and p40, respectively (Kourko *et al*, 2019). While p28 exhibits a classical four‐α helical fold, EBI3 is structurally similar to soluble cytokine receptors (Rousseau *et al*, 2010). Unlike other heterodimeric cytokines (IL‐12 and IL‐23) which activate Signal Transducer and Activator of Transcription (STAT) STAT3 and STAT4, IL‐27 primarily induces activation of STAT1 and STAT3 pathways (Tait Wojno *et al*, 2019). These differences can be attributed to the engagement of GP130 by IL‐27, which is shared across the IL‐6 cytokine family (Trinchieri *et al*, 2003).

IL‐27 bridges two major cytokine families, raising the question of whether competition or synergism could occur in dimer formation between them. Under some conditions, p28 and EBI3 can be secreted independently and associate with other proteins to induce differential responses (Crabe *et al*, 2009; Stumhofer *et al*, 2010; Collison *et al*, 2012; Wang *et al*, 2012; Garbers *et al*, 2013). Promiscuity in chain combinations is characteristic of the IL‐6/IL‐12 family and allows the system to produce different biologically active factors starting from relatively few precursor molecules.

GP130 binds a range of cytokines to elicit diverse cellular responses (Grotzinger *et al*, 1997; Hunter & Jones, 2015). The receptor has two cytokine‐binding sites. The first is located within the elbow between its cytokine homology regions (CHR) and has been shown to engage site 2 of cytokines, including IL‐6 (Boulanger *et al*, 2003b), Leukemia inhibitory factor (LIF; Boulanger *et al*, 2003a), and Ciliary neurotrophic factor (CNTF; Skiniotis *et al*, 2008). The N‐terminal immunoglobulin (Ig) domain of GP130 comprises an additional cytokine‐binding interface that recognizes an epitope at site 3, resulting in higher order assemblies with different stoichiometries (Boulanger *et al*, 2003b; Wang *et al*, 2009). In the hexameric GP130:IL‐6 signaling complex, two molecules of GP130 dimerize to trigger JAK/STAT activation (Wang *et al*, 2009). Here, the two low affinity site 3 interfaces are necessary to stabilize the complex and trigger receptor activation by IL‐6. For GP130 complexes with LIF and CNTF cytokines, site 3 is occupied by the co‐receptor LIF‐R (Huyton *et al*, 2007; Skiniotis *et al*, 2008). Structural details for how GP130 forms a heterodimeric signaling complex by binding site 3 of a cytokine remains unresolved.

Here, we report a 4.0 Å resolution cryo‐EM structure of IL‐27 in complex with cytokine binding domains of GP130 (domains 1–3) and its co‐receptor IL‐27Rα (domains 1–2). This structure reveals a modular assembly mechanism that differs from those described for other members of the IL‐12 and IL‐6 families. IL‐27 binds with high affinity to IL‐27Rα at site 2, which is further stabilized by electrostatic interactions between IL‐27Rα and EBI3. GP130 is recruited to site 3 of the heterodimeric cytokine. Our structural and biochemical data explain how IL‐27 coordinates two signaling receptors to modulate T cell‐mediated inflammation. Our results provide a blueprint for developing new therapeutics that target and tune IL‐27 responses.

## Results and Discussion

### Molecular architecture of the IL‐27 cytokine recognition complex

IL‐27 signals through dimerization of the cognate receptor IL‐27Rα and the shared receptor GP130 (Wilmes *et al*, 2021). Current models for the assembly of complexes mediated by IL‐27 have been largely based on structural principles derived from the IL‐12/IL‐23 or IL‐6 systems. However, given differences in signaling outcomes between IL‐27 and these two families, together with the growing importance of IL‐27 as a therapeutic target, we used cryoEM to solve the structure of the IL‐27 cytokine recognition complex.

**Figure 1:**
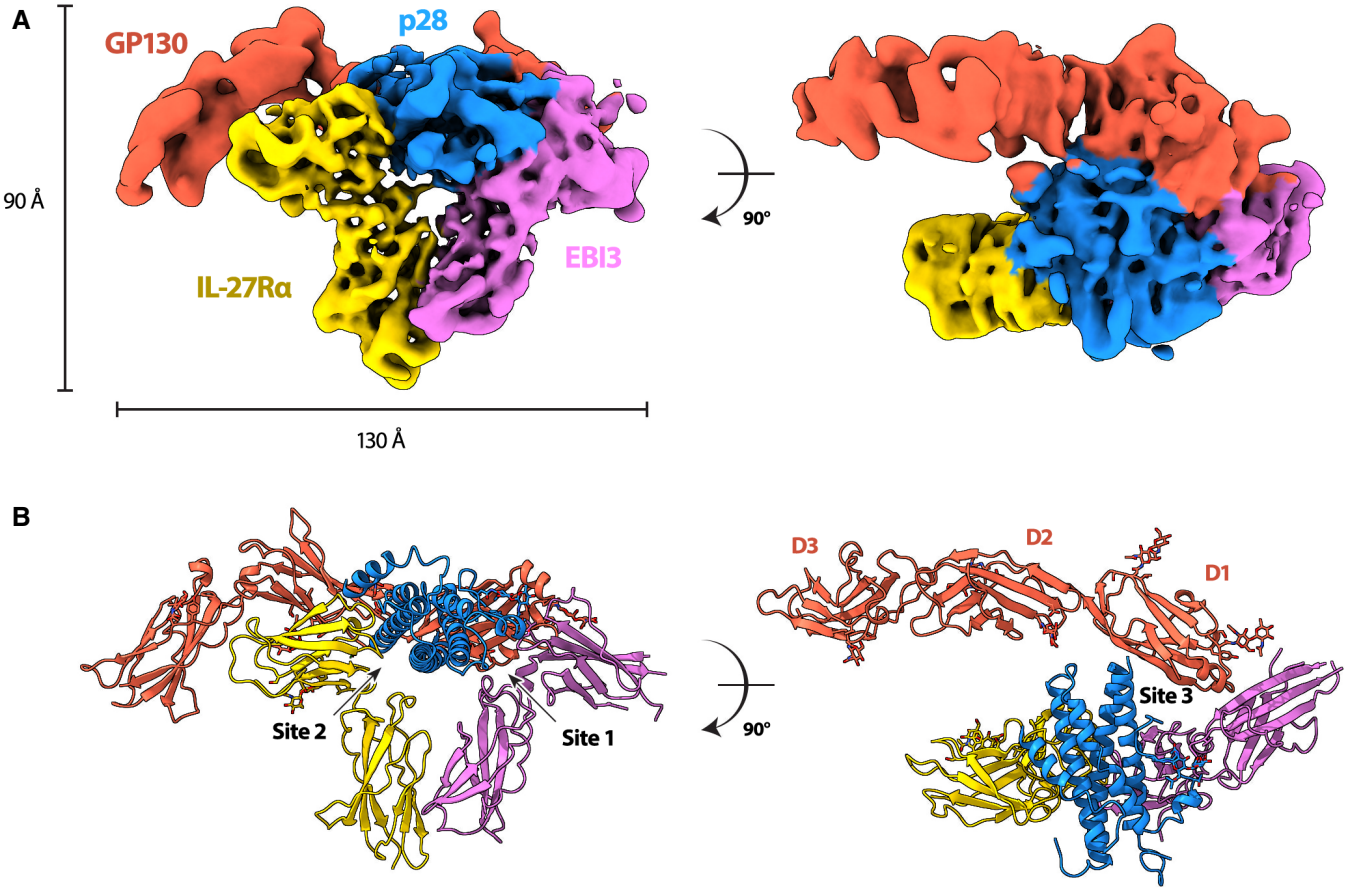
Structure of the IL‐27 receptor recognition complex A, B
CryoEM reconstruction (A) and atomic model (B) of the IL‐27 receptor recognition complex. The complex consists of the IL‐27 heterodimeric cytokine, p28 (blue) and EBI3 (purple), bound to two signaling receptors, GP130 (red) and IL‐27Rα (yellow). EBI3 occupies site 1 on p28, while IL‐27Rα occupies site 2. GP130 binds p28 at site 3 via domains 1 and 2 (D1, D2). The canonical cytokine recognition site on GP130 between domains D2 and D3 is unoccupied.

**Figure EV1:**
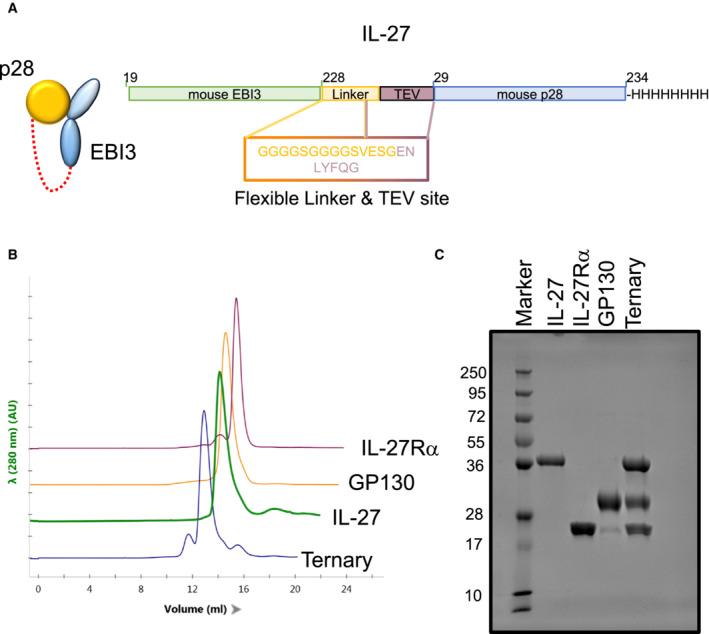
Assembly and characterization of the IL‐27‐receptor complex A
Schematic representation of the single chain IL‐27 heterodimer.B
Chromatogram overlay from size exclusion chromatography of the different IL‐27 complex components.C
Coomassie‐stained SDS‐PAGE analysis under reducing conditions of the individual components plus the IL‐27:IL‐27Rα:GP130 complex used for cryoEM studies.

**Figure EV2:**
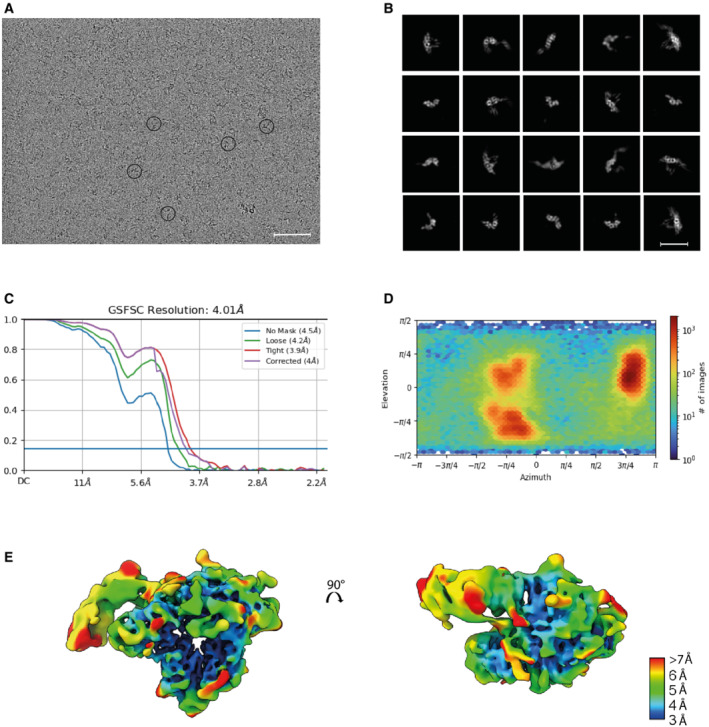
Supplementary cryoEM data A
Raw cryoEM micrograph with examples of the IL‐27 receptor complex circled. Scale bar 50 nm.B
A subset of representative 2D class averages. Scale bar 110 Å.C
Gold standard Fourier shell correlation (GSFSC) for the final reconstruction. The resolution at the 0.143 cutoff is reported.D
Angular distribution plot.E
IL‐27 receptor recognition reconstruction filtered according to local resolution ranging from 3 Å (blue) to 7–15 Å (red).

**Figure EV3:**
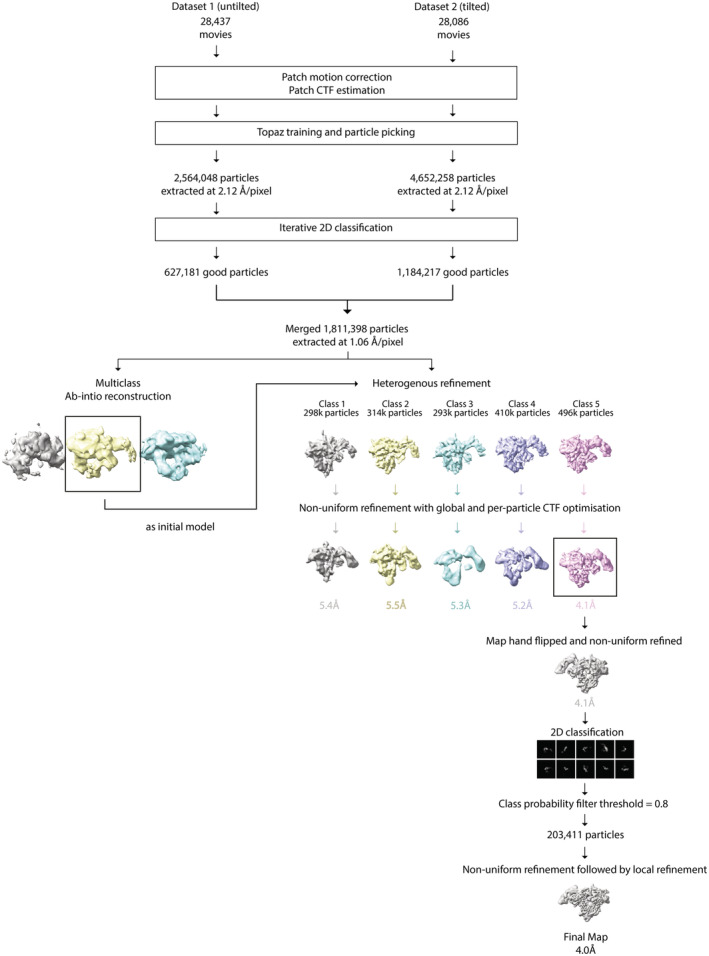
CryoEM image processing workflow outlining steps performed to obtain the structure of IL‐27 heterodimer complex All processing was performed using CryoSPARC v.3.3.1 (see Materials and Methods for details).

**Figure EV4:**
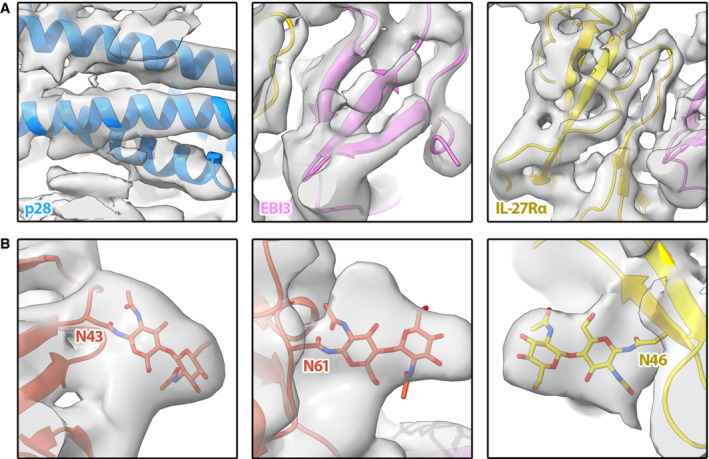
Model for the IL‐27 receptor signaling complex overlaid with the cryoEM map A
Map/model overlays for density corresponding to p28 (left panel), EBI3 (middle panel), and IL‐27Rα (right panel).B
Representative glycan density in the map corresponding to known glycosylation sites on GP130 (left and middle panels) and IL‐27Rα (right panel).

To overcome challenges in cytokine stability, we engineered an IL‐27 cytokine variant in which the two monomeric components (EBI3 and p28) were fused by a short linker (Wilmes *et al*, 2021; Fig EV1A). The fusion cytokine was expressed in insect cells and purified together with domains 1–3 (D1, D2, D3) of GP130 and the first two domains of IL‐27Rα (Fig EV1B and C). We then used cryoEM to solve the structure of the complete IL‐27 cytokine‐recognition complex (Figs 1A and EV2A–C). To address preferred orientation issues of the sample, we collected and merged datasets recorded at 0, 30, and 35 degrees along the tilt axis of the microscope (Fig EV2D). We used the ab initio reconstruction protocols within cryoSPARC (Punjani *et al*, 2017) to generate an initial model of the complex. Maps were further refined using a combination of nonuniform and heterogeneous refinement procedures (Fig EV3). The final map was refined to a reported resolution of 4.0 Å using the gold standard FSC 0.143 cut‐off (Fig EV2C); with local resolution ranging from 3.5 to 15.1 Å (Fig EV2E). In our map, individual helices of p28 and β‐strands of both EBI3 and IL‐27Rα are clearly resolved (Fig EV4A). The chirality of the helical bundle central to the p28 cytokine was used to assign the handedness of the reconstruction. Although density for GP130 was less well ordered, we observe density for all three domains including the low‐density core of the CHR domains, which guided placement of GP130 as a single rigid body. Given the lack of side‐chain density at this resolution, models were derived from Alphafold predictions (Jumper *et al*, 2021) refined with strong adaptive distance restraints and geometric restraints imposed (Table EV1). While there may be some uncertainty in the side‐chain rotamer, interface residues are defined largely by the rigid‐body fit of AlphaFold models.

The overall structure of the IL‐27 cytokine recognition complex exhibits a classical architecture with a helical cytokine bundle central to the assembly (Fig 1B). For the IL‐27 complex, the helical bundle is sandwiched by two ‘L'‐shaped densities and an additional prong that contacts the back face of the heterodimeric cytokine. Our data agrees with a recently published structure of the human IL‐27 signaling complex (Caveney *et al*, 2022), with slight differences in rotation of the receptors relative to the helical cytokine.

### Principle binding interfaces for IL‐27 cytokine recognition

**Figure 2:**
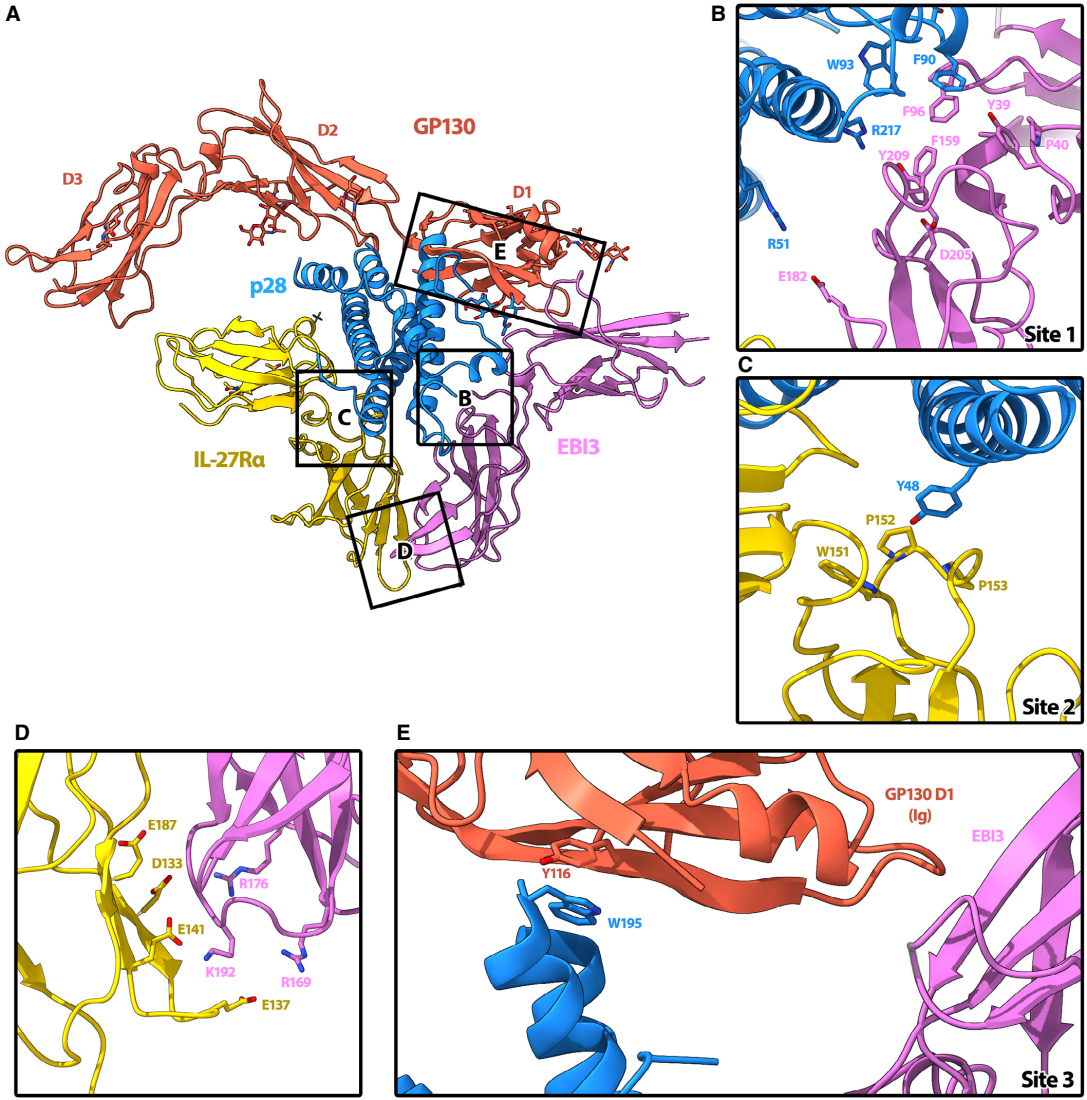
Interaction interfaces of the complex A
Overview of IL‐27 interactions with signaling receptors. Ribbon representation of the complex colored according to protein components: p28 (blue), EBI3 (purple), IL‐27Rα (yellow), GP130 (red). Individual domains of GP130 (D1, D2, and D3) are labeled.B
The hinge between the two CHR domains of EBI3 form a hydrophobic groove (Y39, P40, F96, F159, and Y209) that is filled by W93 of p28. Residue EBI3: D205, which is important for assembly of the heterodimeric cytokine (Rousseau *et al*, 2010), is also present in the binding interface and could form a salt bridge with p28:R217.C
IL‐27Rα binds site 2 of p28 at the apex of the elbow between its two CHR domains. The loops of IL‐27Rα form a pocket comprised of residues IL‐27Rα: W151, P152, and P153 in which the aromatic side chain of p28:Y48 could slot into.D
The orientation of IL‐27Rα at site 2 is stabilized by a second interaction interface with EBI3, which is dominated by electrostatic complementarity between the two domains (IL‐27Rα: E187, D133, E141, E137, and EBI3:R176, R169, K192).E
Site 3 of p28 is occupied by the bend between the CHR domain 2 (D2) and the immunoglobulin (Ig) domain D1 of GP130. While this interface is not well resolved, p28:W195, which is essential for GP130‐mediated signaling (Rousseau *et al*, 2010), is facing D1. The key residues that mediate the interactions at interfaces are shown as sticks.

Cytokines from the IL‐12/IL‐6 family have three highly‐conserved principle binding interfaces: site 1, site 2, and site 3 (Wang *et al*, 2009). For the IL‐27 recognition complex, site 1 is occupied by the second half of the heterodimeric cytokine, EBI3 (Fig 2A and B). EBI3 is comprised of two Fibronectin type III (FNIII) domains bent into an ‘L'‐shaped arrangement. Docking and minimal refinement of the EBI3 Alphafold model into the map show that the bend of the EBI3 elbow is formed by a cluster of aromatic residues (EBI3: Y39, F96, F159, and Y209) together with a proline (EBI3:P40), which create a hydrophobic groove recognized by p28:W93 (Fig 2B). This feature of knobs and holes shape recognition is consistent with other cytokine‐binding interfaces (Wang *et al*, 2009). Our p28:EBI3 interface is supported by mutagenesis studies showing that equivalent residues in the human cytokine (EBI3:F97; p28:W97) are essential for cytokine complex formation (Rousseau *et al*, 2010). The same study identified an aspartic acid on EBI3 that also influences stability of the heterodimer (Rousseau *et al*, 2010). In our model, the equivalent murine residue (EBI3:D205) is nearby an arginine on p28 (p28:R217) and may be important for maintaining the interface (Fig 2B).

For the IL‐6:GP130 cytokine recognition complex, site 2 of IL‐6 is occupied by the two CHR domains of GP130 (D2 and D3; Boulanger *et al*, 2003b). To understand how GP130 recognizes chemically unique cytokines, we next investigated the IL‐27 site 2 interface. In contrast to the IL‐6 recognition complex, we find that site 2 of p28 is formed by the apex of the elbow region between the two CHR domains of IL‐27Rα (Fig 2A and C). Here, we find that the knob and hole pattern is encoded by cluster of proline residues (IL‐27Rα: P153, P152) and an aromatic residue (W151) of IL‐27Rα, which form a pocket to bind a tyrosine extending from p28 (p28:Y48; Fig 2C). In the IL‐6 complex, the equivalent tyrosine on IL‐6 slots into a hydrophobic groove formed by the interface between D2 and D3 of GP130 (Boulanger *et al*, 2003b).

**Figure EV5:**
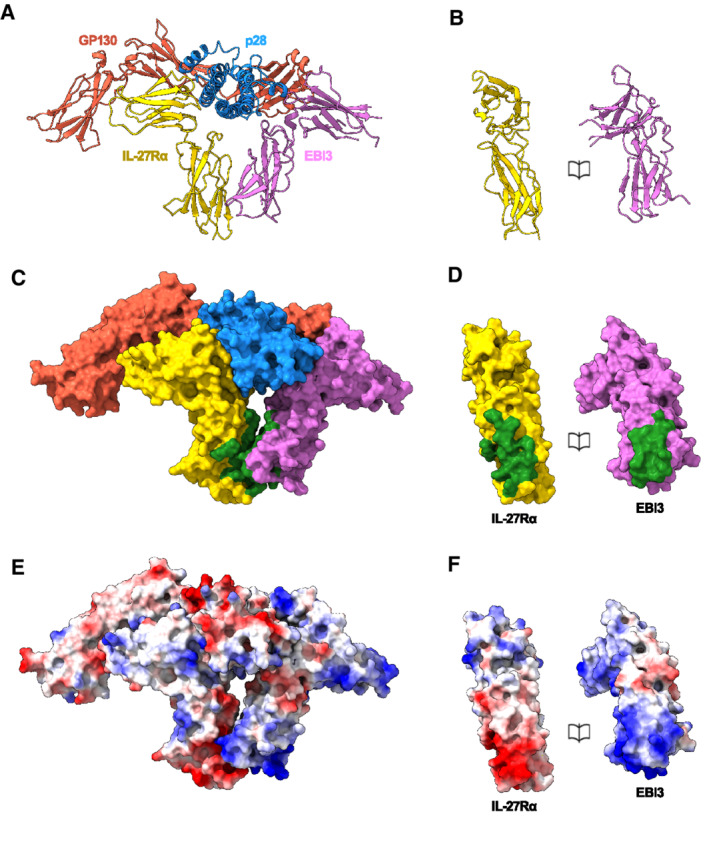
EBI3:IL‐27Rα interface A–F
Ribbon representation of the IL‐27 receptor recognition complex (A) and book representation of the EBI3:IL‐27Rα interface (B). Individual proteins colored: EBI3 (purple), IL‐27Rα (yellow), p28 (blue), GP130 (red). Surface representation of the complex (C) and book representation of the interface (D). Proteins colored as in (A) with EBI3: IL‐27Rα interface residues in green. Coulombic electrostatic potential ranging from −10 (red) to 10 (blue) kcal/(mole) calculated from the models in A (E) and corresponding book representation of the interface (F).

The position of EBI3 at site 1 and IL‐27Rα at site 2 is coordinated by a second interaction interface. EBI3 directly contacts the second CHR domain of IL‐27Rα. By contrast to the hydrophobic knob and hole recognition motifs observed for p28, this secondary interface is dominated by electrostatic interactions (Figs 2A and D, and EV5A–E). Positively charged residues of EBI3 (EBI3: R169, R176, K192) face a negatively charged surface of IL‐27Rα (IL‐27Rα: D133, E137, E141, E187). These residues could be involved in a series of salt bridges that further contribute to the stability of the assembly.

The three N‐terminal domains of GP130 are essential for cytokine recognition and signaling (Dahmen *et al*, 1998; Boulanger *et al*, 2003b). A crystal structure of GP130 in complex with IL‐6 showed that GP130 can engage cytokines through both site 2 and site 3 interactions (Boulanger *et al*, 2003b). While the first two CHR domains of GP130 (D2 and D3) form the primary interface at site 2, the Ig domain (D1) of GP130 at site 3 bridges a second cytokine and is responsible for the lateral hexameric complex. In our structure of the IL‐27 cytokine recognition complex, we observe a third prong of density extending from the core complex consistent with the arrangement of D1–D3 in the GP130 crystal structure (Boulanger *et al*, 2003b). In the IL27‐receptor signaling complex, D1 of GP130 contacts p28 at site 3 and bridges EBI3 in the heterodimeric cytokine (Fig 2A and E). Specifically, the aromatic side chain of p28 W195 could pack against GP130 Y116. Indeed, biochemical data confirm that GP130 engages p28 at site 3 with the equivalent residue in the human p28 (W197) playing a key role (Rousseau *et al*, 2010).

**Figure 3:**
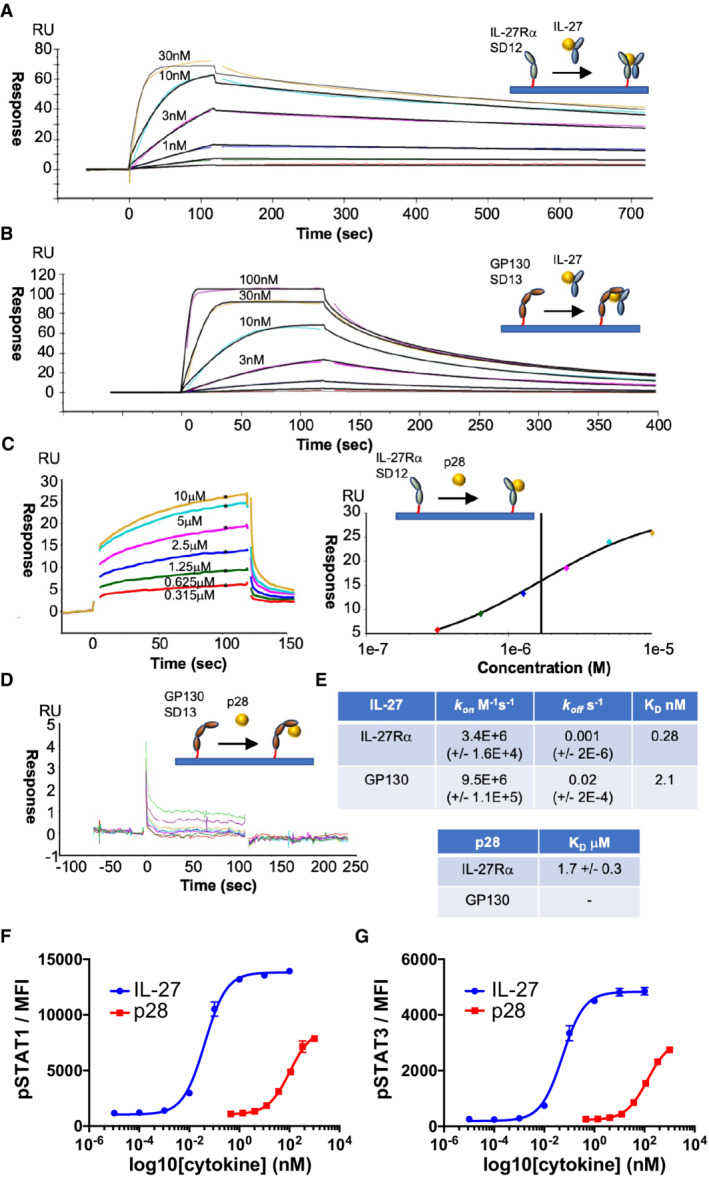
Biophysical analysis of the assembly of the IL‐27 receptor complex A–E
For SPR measurements, IL‐27Rα or GP130 was immobilized on the chip surface by biotin–streptavidin interaction and IL‐27 or p28 was flowed across the chip in solution. SPR data are representative of three biological replicates. Kinetic charts for IL‐27Rα (A) and GP130 (B). Concentrations used are shown on the curves. Data traces were fitted using a 1:1 interaction model (black) to quantify the kinetics (k_on_, k_off_) and binding affinity (K_D_) of the interactions. (C, D) Equilibrium chart for p28 binding to IL‐27Rα (C, left panel) and curve‐fitting to data points generated at various concentrations of p28 (C, right panel). (D) Equilibrium chart for p28 binding GP130. (E) Table presenting kinetically derived (IL‐27) and thermodynamically derived (p28) binding constants. Standard Error values (SEs) are shown in parentheses.F, G
Dose response curves for pSTAT1 (F) and pSTAT3 (G) in resting mouse CD8^+^ T cells. Cells were stimulated with mIL‐27 or p28 for 15 min with the indicated doses. Data shown are the mean of four biological replicates with error bars depicting standard error of the mean.

### Kinetic drivers of IL‐27 signaling complex assembly

To understand the kinetic drivers underpinning assembly of the IL‐27 signaling complex, we defined the binding affinities for each of the subcomponents using surface plasmon resonance (SPR; Fig 3A–E). First, we set out to identify which of the two signaling receptors bound the IL‐27 heterodimer with higher affinity. We immobilized biotinylated IL‐27Rα or GP130 on a streptavidin SPR surface and passed a range of IL‐27 concentrations to measure rates of association and dissociation (Fig 3A and B). We found that IL‐27Rα has a higher affinity (K_D_: 0.28 nM) for the heterodimeric cytokine than GP130 (K_D_: 2.1 nM; Fig 3E), in agreement with biochemical data showing that GP130 engages the low‐affinity binding site 3 (Rousseau *et al*, 2010). These results suggest a two‐step binding mode for IL‐27 complex formation, where IL‐27 binds IL‐27Rα with high affinity in a first step, and subsequently recruits GP130 with lower affinity to form the signaling complex. We next wanted to understand the roles of EBI3 and p28 in the kinetics of complex formation. As we were unable to produce recombinant EBI3, we focused our analysis on p28. We quantified the binding affinity of p28 for each signaling receptor immobilized to the streptavidin SPR chip (Fig 3C and D). We observed only weak binding of p28 to IL‐27Rα (K_D_: 1.7 μM) and no binding to GP130 at the concentrations tested (Fig 3E). These data are supported by our structural observations that the GP130 binding site is formed by the cleft between p28 and EBI3 and are consistent with weak signaling responses elicited by p28 when compared to IL‐27 in CD8 T cells (Fig 3F and G). Taken together, these data support a stabilizing role of EBI3 in formation of the IL‐27 signaling complex.

**Figure 4:**
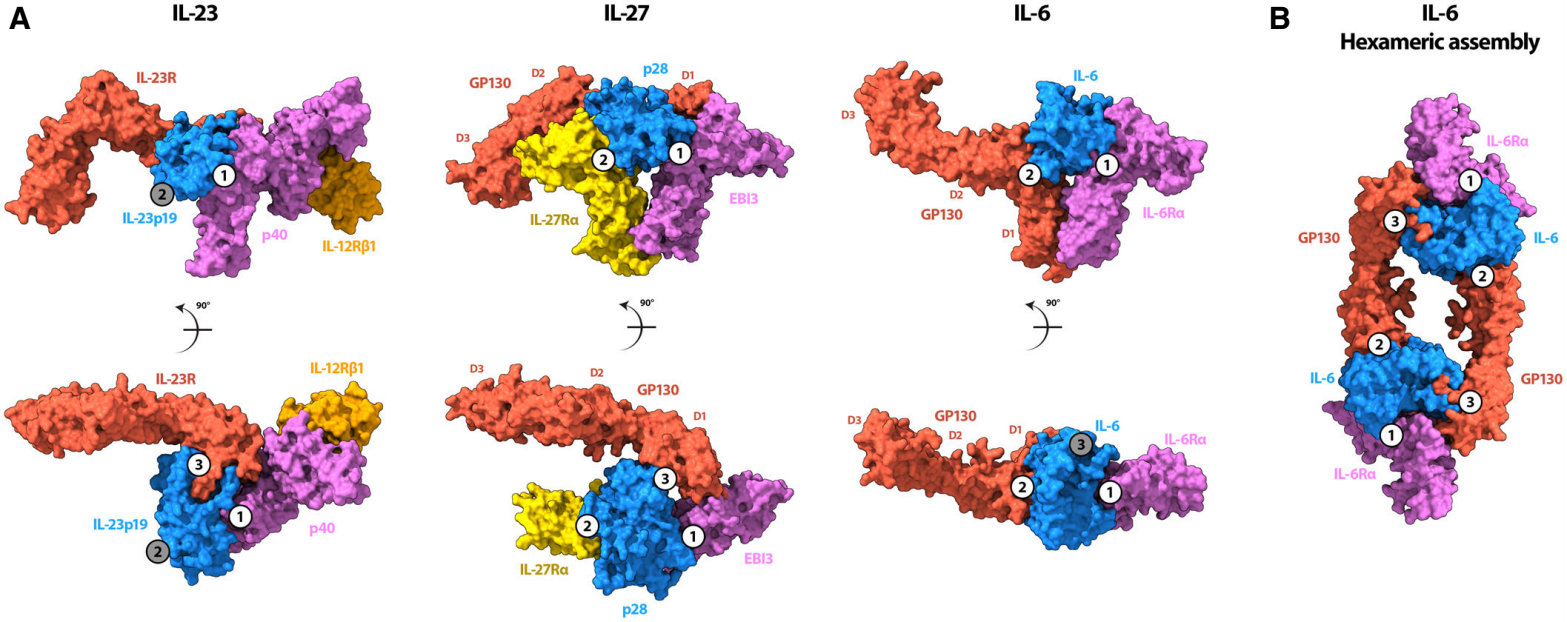
Structural comparison of IL‐27 receptor recognition complex with IL‐23 and IL‐6 cytokine families A
(left panel) Surface rendering of the IL‐23 receptor recognition complex (Data ref: Glassman *et al*, 2021a) colored according to protein components: IL‐23 receptor (red), IL‐12β (p40) (purple), IL‐23p19 (blue), IL‐12Rβ1 (orange). A comparison with IL‐27 receptor complex (middle panel) shows that both GP130 (red) and IL‐23 receptor occupy site 3 of their respective cytokines, orienting domains 2 and 3 similarly towards the membrane. Both EBI3 (purple) and the other component of the IL‐23 heterocytokine, p40, occupy the site 1 epitope. Surface rendering of the IL‐6 receptor recognition complex (Data ref: Boulanger *et al*, 2003c) colored according to protein components: IL‐6 (blue), IL‐6Rα (purple), GP130 (red; right panel). A comparison with the IL‐27 receptor complex shows that the nonsignaling components of both complexes (EBI3 and IL‐6Rα) occupy site 1, while GP130 binds in a different way. For IL‐6, GP130 binds site 2 through an interaction site located between the CHR domains, D2 and D3. Instead, GP130 binds site 3 of the IL‐27 cytokine between domains D1 and D2, leaving the canonical cytokine binding site unoccupied.B
Surface rendering of the hexameric IL‐6 assembly (PDB: 1P9M) colored according to protein components: IL‐6 (blue), IL‐6Rα (purple), GP130 (red). GP130 bound to site 2 of one IL‐6 molecule bridges a second cytokine by binding at site 3 to stabilize the complex. Data information: Sites 1, 2, and 3 are indicated as numbered circles in each of the panels. Occupied sites are shown as white circles, while unoccupied sites are gray.

IL‐27 is a model system for signaling by heterodimeric cytokines, including IL‐12 and IL‐23. A comparison of the heterodimeric IL‐23 receptor complex (Data ref: Glassman *et al*, 2021a; Glassman *et al*, 2021b) with our structure shows that EBI3 and the IL‐12β subunit (p40) overlay with site 1 (Fig 4A). In addition, we observe that the GP130 density extending away from the core IL‐27 complex has similarities to the IL‐23 signaling receptor. The arrangement of these extracellular domains likely directs the orientation of intracellular regions that activate the JAK/STAT pathway. Therefore, it may be possible that the bend between D2 and D3 of GP130 in the signaling receptor is a topological requirement for activation by IL‐12, IL‐23, and IL‐27.

IL‐27 is a central member of the IL‐6 family of cytokines that signal through GP130 (Hunter & Kastelein, 2012). The diversity in cellular responses within the family stems in part from the strict transcriptional control of cytokines secreted by different cell types (Taga & Kishimoto, 1997). Although IL‐27 is a heterodimeric cytokine, there are some instances whereby the two components are independently expressed (Devergne *et al*, 1996; Maaser *et al*, 2004). In the absence of EBI3, p28 can act as an antagonist for IL‐6‐mediated GP130 signaling (Stumhofer *et al*, 2010). Our biophysical data show that binding of p28 alone to GP130 is negligible (Fig 3). A comparison of the IL‐6 cytokine recognition complex (Data ref: Boulanger *et al*, 2003c) with our structure shows that binding of EBI3 to site 1 overlaps with site 1 of the IL‐6 (Fig 4A). Previous studies have reported that p28 can bind IL‐6Rα (Garbers *et al*, 2013). Though signaling by the putative p28:IL‐6Rα is weaker compared to IL‐6 or IL‐27, it may be possible that IL‐6Rα engages p28 at site 1 with low affinity to produce weak agonistic or antagonistic activities in certain contexts.

Chain sharing is a common theme among cytokine receptors (Vignali & Kuchroo, 2012) and contributes to the layers of complexity derived from relatively few building blocks. Although both IL‐27 and IL‐6 engage GP130, they do so through different interaction interfaces. One important difference between these two structures occurs at site 2 (Fig 4A). While the two CHR domains of GP130 (D2 and D3) bind IL‐6 at site 2, the equivalent position in the IL‐27 system is occupied by IL‐27Rα. Unlike the IL‐6 co‐receptor (IL‐6Rα), the intracellular domains of GP130 and IL‐27Rα both associate with JAKs proteins and activate signaling upon dimer formation. In the case of IL‐27, the cognate co‐receptor IL‐27Rα could fill an analogous role to GP130 at site 2 in the IL‐6‐signaling complex. In both systems, site 1 is occupied by nonsignaling proteins: EBI3 and IL‐6Rα. Our structural and biophysical data support role for auxiliary proteins at site 1 to enhance the stability of the signaling complex, while receptors occupying site 2 are responsible for signal transduction.

Signaling through the JAK/STAT pathway requires dimerization of signaling receptors. How that dimerization is achieved, and how the geometry of the signaling complex contributes to the functional diversity of the cascade, remains an open question. There is a wide range of oligomeric assemblies observed for GP130‐cytokine complexes (Boulanger *et al*, 2003b; Wang *et al*, 2009). For IL‐6, dimerization of GP130 occurs through a hexameric assembly (2 copies of the IL‐6:IL‐6Rα:GP130 complex). Within the hexamer, GP130 bound to IL‐6 site 2 dimerizes with a second copy bound to site 3 of the additional cytokine (Fig 4B). In the IL‐27 signaling complex, dimerization of IL‐27Rα and GP130 may occur from receptors occupying sites 2 and 3 from the same cytokine, though additional cytokine‐receptor stoichiometries cannot be ruled out. Indeed, the primary interaction interface observed in the IL‐6:GP130 complex occurs at the apex of a bend between GP130 CHR domains (D2 and D3). In the IL‐27 complex, this region is likely unoccupied and available to engage other cytokines. In addition, IL‐27 has been shown to inhibit signaling by oncostatin M (OSM), an IL‐6 family cytokine (Baker *et al*, 2010). Based on our structure, IL‐27 immunosuppressant activities may be achieved by blocking inflammatory cytokines such as OSM from binding GP130 at site 2 in a signaling competent conformation. Understanding how mixtures of cytokines influence signaling assemblies and downstream cascades will inform new strategies in protein engineering for fine‐tuning cytokine responses.

## Materials and Methods

### Protein expression and purification

Murine IL‐27 was cloned as a linker‐connected single‐chain variant (p28 + EBI3) as described in Oniki *et al* (2006) (Fig EV1A). In the text, we refer to this variant only as IL‐27. Murine IL‐27Rα (amino acids 28–224) and GP130 (amino acid 23–319) ectodomains were cloned and expressed as described in Martinez‐Fabregas *et al* (2019). Briefly, protein sequences were cloned into the pAcGP67‐A vector (BD Biosciences) in frame with an N‐terminal gp67 signal sequence, driving protein secretion, and a C‐terminal hexahistidine tag. Baculovirus stocks were produced by transfection and amplification in *Spodoptera frugiperda* (*Sf*9) cells grown in SF900III media (Invitrogen), and protein expression was carried out in suspension *Trichoplusia ni* (High Five) cells grown in InsectXpress media (Lonza).

Protein purification was carried out using the method described in Spangler *et al* (2019). Hi‐Five cells were pelleted with centrifugation at 1,000 × *g*, and impurities from the remaining media were removed by a precipitation step through addition of Tris pH 8.0, CaCl_2_, and NiCl_2_ to final concentrations of 200, 50, and 1 mM, respectively. The precipitate formed was then removed through centrifugation at 9,000 × *g*. Nickel‐NTA agarose beads (Qiagen) were added to the clarified media and the target proteins purified through batch binding followed by column washing in HBS buffer for murine IL‐27Rα and GP130 or HBS‐Hi buffer (HBS buffer supplemented to 500 mM of NaCl and 5% glycerol, pH 7.2) for murine IL‐27sc. Elution was performed using HBS or HBS‐Hi buffer plus 200 mM imidazole. Final purification was performed by size exclusion chromatography on an ENrich SEC 650 10 × 300 column (Biorad), equilibrated in HBS or HBS‐Hi buffers. Concentration of the purified sample was carried out using 30 kDa Millipore Amicon‐Ultra spin concentrators. Recombinant proteins were purified to greater than 98% homogeneity. For cryoEM studies, IL‐27Rα, GP130, and IL‐27 were mixed in a 1:1:1 ratio and subsequently purified by size‐exclusion chromatography.

To generate biotinylated proteins for surface plasmon resonance studies, the GP130 sequence was subcloned into the pAcGP67‐A vector with a C‐terminal biotin acceptor peptide (BAP)–LNDIFEAQKIEWHW followed by a hexa‐histidine tag. The purified protein was biotinylated with BirA ligase following a previously described protocol (Spangler *et al*, 2019). IL‐27Rα was N‐terminal biotinylated *in vitro* using EZ‐Link Sulfo‐NHS‐SS‐Biotin (Pierce) at pH 6.5.

### Surface plasmon resonance

Surface plasmon resonance experiments were performed to determine the binding affinity of the recombinantly produced IL‐27 and p28 to IL‐27Rα and GP130. These were carried out on a Biacore T100 instrument (T200 sensitivity enhanced). C‐terminal biotinylated mouse GP130 and mouse IL‐27Rα were immobilized onto a SA sensor chip (GE Healthcare) at levels of ~100 response units (RU). The immobilization was performed in 10 mM HEPES, 150 mM NaCl, 0.02% (v/v) TWEEN‐20, pH 7.2 buffer. Analysis runs were performed at 25°C in 10 mM HEPES, 150 mM NaCl, 0.05% (v/v) TWEEN‐20, pH 7.2, and 0.5% BSA. Mouse IL‐27 binding was tested by injecting a 1:3 dilution series of inhibitor from a top concentration of 100 nM, and at a flow rate of 30 μl/min. Mouse p28 binding was tested in a similar manner, but from a top concentration of 20 μM and with a 1:2 dilution series. In both cases, the association time was set to 120 s, while the dissociation time was 600 s for IL‐27 and 60 s for p28. Data analysis was performed using Biacore T200 Evaluation Software 3.0.

### Phospho‐flow analysis

For phospho‐flow analysis of STAT1 and STAT3, mouse CD8 T cells were purified from a wild‐type spleen, kindly provided by the Cantrell's laboratory (Dundee), and were plated at a density of 2 × 105 cells per well in 50 μL in a 96‐well V bottom plate. Cells were left unstimulated or simulated with 3‐fold serially diluted IL‐27sc or p28 variants (50 μL per well) for 15 min at 37°C before fixation with 2% paraformaldehyde for 10 min at room temperature. Cells were washed in PBS and permeabilized in ice‐cold 100% methanol and incubated on ice for a minimum of 30 min. Cells were fluorescently barcoded as previously described in Krutzik *et al* (2006). Briefly, a panel of 16 combinations of two NHS dyes (Pacific Blue and DyLight800, Thermo) was used to stain individual wells on ice for 35 min before stopping the reaction by washing in PBS/0.5% BSA. Once barcoded, the 16 populations were pooled together for antibody staining. Cells were stained with anti‐pSTAT3Alexa488 (Biolegend #651006) and anti‐pSTAT1Alexa647 (Cell Signaling Technologies #8009). During acquisition, individual populations were identified according to the barcoding pattern and pSTAT3Alexa488 and pSTAT1Alexa647 MFI was quantified for all populations. MFI was plotted and sigmoidal dose response curves were fitted using Prism software (Version 7, GraphPad). Mice were maintained in the Biological Resource Unit at the University of Dundee using procedures that were approved by the University Ethical Review Committee and under the authorization of the UK Home Office Animals (Scientific Procedures) Act 1986.

### CryoEM sample preparation and data collection

Preformed IL‐27:IL‐27Rα:GP130 complex sample was diluted to 0.1 mg/ml and vitrified in liquid ethane using Vitrobot Mark IV (Thermo Fisher Scientific). Lacey Carbon Au 300 grids (EMS) were glow discharged in air using a Cressington 208 for 60 s prior to application of the sample. Four microlitre of sample were applied to the grids at a temperature of 20°C and a humidity level of 100%. Grids were then immediately blotted (force −2, time 2 s) and plunge‐frozen into liquid ethane cooled to liquid nitrogen temperature.

Grids were imaged using a 300 kV Titan Krios transmission electron microscope (Thermo Fisher Scientific) equipped with K3 camera (Gatan) operated in super resolution mode. Movies were collected at 81,000× magnification and binned by two on the camera (calibrated pixel size of 1.06 Å/pixel) with a total dose of 50 electrons per Å^2^ using EPU (Thermo Fisher Scientific, version 2.11.1.11) automated data software. The first dataset was collected at 0 degree tilt and consisted of 28,437 image stacks taken over a defocus range of −0.5 to −2.25 μm in 0.25 μm increments. As initial processing of the first dataset showed that the particles adopted a favored orientation, we used the cryoEF software to estimate the appropriate tilt angle for data collection and acquisition of missing views. Although the recommended tilt angle reported by cryoEF (Naydenova & Russo, 2017) was 37°, due to sample drift at high tilt, we collected 6,612 image stacks at 35° and 21,474 image stacks at 30°. Tilted images were taken over a defocus range of −1.5 to −2.7 μm in 0.3 μm steps. Movie stacks were collected with similar conditions as dataset‐1. A summary of imaging conditions is presented in Table EV1.

### CryoEM data processing

Dataset‐1, which comprised 28,437 micrographs, was pre‐processed using cryoSPARC (v.3.3.1; Punjani *et al*, 2017) patch motion correction and patch CTF. The micrographs were manually curated to remove movies with substantial drift and crystalline ice, resulting in a total of 17,228 micrographs going on for further processing. Deep learning models used in Topaz (v.0.2.5; Bepler *et al*, 2020) were used to automatically pick particles from these micrographs within the cryoSPARC workflow. We used the Topaz pre‐trained model (ResnNet16) for particle picking in the first instance. During the initial 2D classification, rare views were combined and used as input for a second round of Topaz training. Particles from both rounds were combined and duplicates were removed. A total of 3,575,367 particles were extracted at 2.12 Å/pixel (bin by 2) and subject to iterative 2D classification to remove ice contamination, carbon edges and broken particles, resulting in a total of 627,181 particles from dataset‐1.

Dataset‐2, which comprised 28,086 tilted image stacks, was preprocessed in cryoSPARC and curated as described for dataset‐1. Of these 18,838 micrographs were used for particle picking in Topaz. A total of 4,652,258 particles were extracted at 2.12 Å/pixel (bin by 2) and subject to iterative 2D classification to remove ice contamination, carbon edges, and broken particles, resulting in a total of 1,184,217 particles from dataset‐2.

Particles from dataset‐1 and dataset‐2 were merged and 1,811,398 particles were re‐extracted at 1.06 Å/pixel and used to create an initial model using cryoSPARC's ab initio reconstruction procedure with three classes. One class showed structural features consistent with the 2D classes and was used as an initial model for further processing. The total dataset (1,811,398 particles) was subjected to heterogeneous refinement with 5 classes. Each of these underwent nonuniform refinement and particles pertaining highest resolution class (496,334 particles) was taken forward. Of these, particles underwent further 2D classification implementing a probability threshold of 0.8 applied to each class. The probability threshold is defined as a value between 0 and 1 and excludes particles with smaller 2D class posterior probabilities compared with probabilities from the particles.alignments2D. The final 203,411 particles went into a round of nonuniform refinement followed by local refinement. All nonuniform refinement runs included optimization of per particle defocus and optimization of per group CTF parameters. The final map resolution 4.0 Å was assessed using the gold standard FSC at a threshold of 0.143 and locally filtered using cryoSPARCs own implementation.

### Model building and refinement

An initial model of IL‐27 was generated by rigid body fitting each protein into the local resolution filtered maps sharpened using a B‐factor of −100. Rigid body fitting was performed in Chimera (Pettersen *et al*, 2004). The models used were murine p28 (AlphaFold Protein Structure Database Q8K3I6, 2022, https://alphafold.ebi.ac.uk/entry/Q8K3I6), murine IL‐27Rα (AlphaFold Protein Structure Database O70394, 2022, https://alphafold.ebi.ac.uk/entry/O70394), murine EBI3 (AlphaFold Protein Structure Database O35228, 2022, https://alphafold.ebi.ac.uk/entry/O35228), and murine GP130 (AlphaFold Protein Structure Database Q00560, 2022, https://alphafold.ebi.ac.uk/entry/Q00560). Alphafold models were trimmed to reflect the domain boundaries in the constructs used and known glycans were added using the carbohydrate module in Coot (Emsley *et al*, 2010; Fig EV4).

The initial model of IL‐27 was refined into the cryoEM map using ISOLDE (v.1.3; Croll, 2018) implemented in ChimeraX (v.1.3; Pettersen *et al*, 2021). During refinement, we applied adaptive distance restraints to each subunit. A loop region of p28 (residues 178–194) was removed to prevent steric clashes with GP130. The atomics models were refined using phenix.real_space_refine in Phenix (v.1.20.1‐4487; Afonine *et al*, 2018) with secondary structure, reference model, and geometry restraints. B‐factors were refined in Phenix. Model FSC validation tools and the overall quality of the model were assessed in the map using the cryoEM validation tools in Phenix and MolProbity (Williams *et al*, 2018; Table EV1). The map‐model FSC was calculated using Refmac5 (Murshudov *et al*, 2011) and reported at a threshold of 0.5. Interface residues were defined by the server Pisa (Krissinel & Henrick, 2007).

## Author contributions

**Yibo Jin:** Formal analysis; investigation; writing – review and editing. **Paul K Fyfe:** Formal analysis; investigation; writing – review and editing. **Scott Gardner:** Formal analysis; investigation; writing – review and editing. **Stephan Wilmes:** Formal analysis; investigation. **Doryen Bubeck:** Conceptualization; formal analysis; supervision; writing – original draft; writing – review and editing. **Ignacio Moraga:** Conceptualization; formal analysis; supervision; funding acquisition; writing – original draft; writing – review and editing.

In addition to the CRediT author contributions listed above, the contributions in detail are:

YJ conducted cryoEM work. SG and DB built and refined atomic models of the complex. PKF recombinantly expressed proteins and perform SPR studies. SW conducted signaling studies. DB and IM conceived the ideas. YJ, SG and DB analyzed cryoEM data. DB and IM wrote the manuscript. SG and YJ generated the figures. All authors assisted with manuscript editing.

## Disclosure and competing interests statement

The authors declare that they have no conflict of interest.

## Supporting information



Expanded View Figures PDF

Table EV1

PDF+

## Data Availability

Data supporting the findings of this manuscript are available from the corresponding authors upon reasonable request. The datasets produced in this study are available in the following databases: i)
IL‐27 receptor complex cryoEM map: Electron microscopy database EMD‐14427 (http://www.ebi.ac.uk/emdb/EMD-14427) ii)
IL‐27 receptor complex structural model: Protein Data Bank PDB‐7Z0L (https://doi.org/10.2210/pdb7Z0L/pdb) accession numbers for the EM map and models of the IL27‐receptor recognition complex reported in this paper are EMD‐14427 and PDB ID 7Z0L.
