# Supplementary material for: Structural insights into the assembly and activation of the IL‐27 signaling complex
Source: EMBO Rep. 2022 Aug 3;23(10):e55450. doi: 10.15252/embr.202255450 (PMC9535766; doi:10.15252/embr.202255450)
Supplement: Supplementary file 1 — Expanded View Figures PDF [file EMBR-23-e55450-s002.pdf]

Expanded View Figures

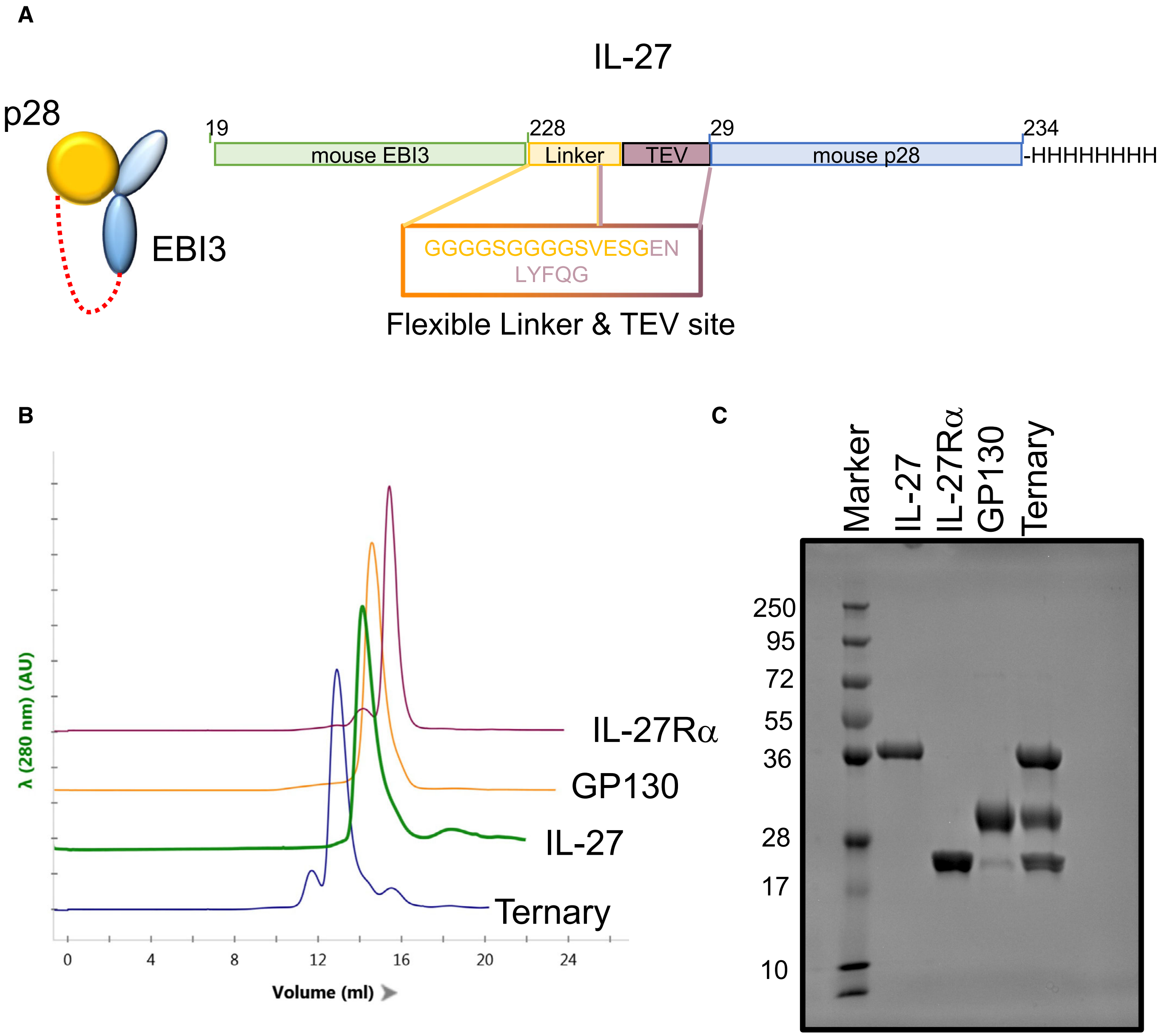

Figure EV1. Assembly and characterization of the IL-27-receptor complex.

A Schematic representation of the single chain IL-27 heterodimer.

B Chromatogram overlay from size exclusion chromatography of the different IL-27 complex components.

C Coomassie-stained SDS-PAGE analysis under reducing conditions of the individual components plus the IL-27:IL-27Rα:GP130 complex used for cryoEM studies.

© 2022 The Authors

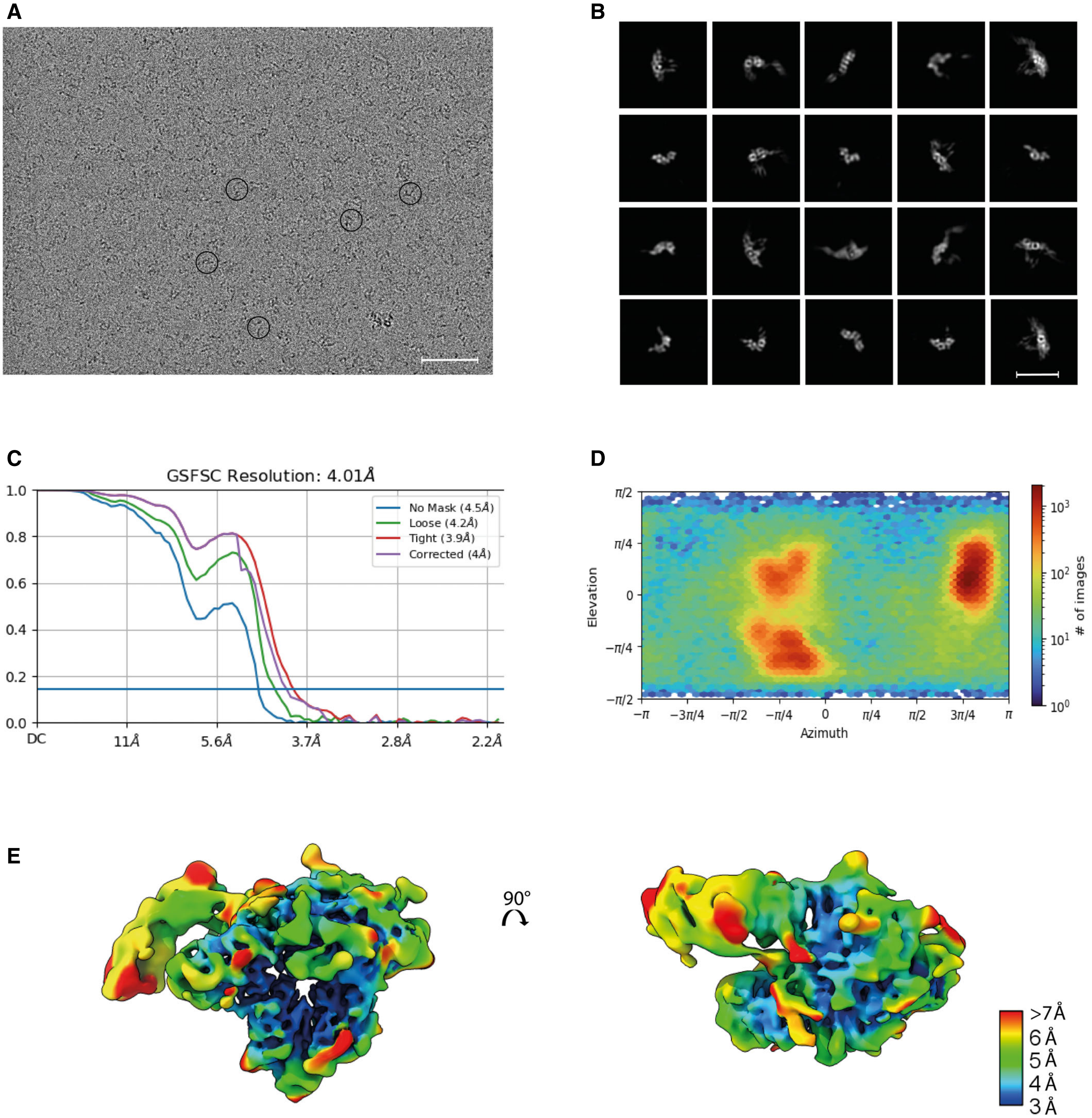

Figure EV2. Supplementary cryoEM data.

A Raw cryoEM micrograph with examples of the IL-27 receptor complex circled. Scale bar 50 nm.
B A subset of representative 2D class averages. Scale bar 110 Å.
C Gold standard Fourier shell correlation (GSFSC) for the final reconstruction. The resolution at the 0.143 cutoff is reported.
D Angular distribution plot.
E IL-27 receptor recognition reconstruction filtered according to local resolution ranging from 3 Å (blue) to 7–15 Å (red).

 © 2022 The Authors

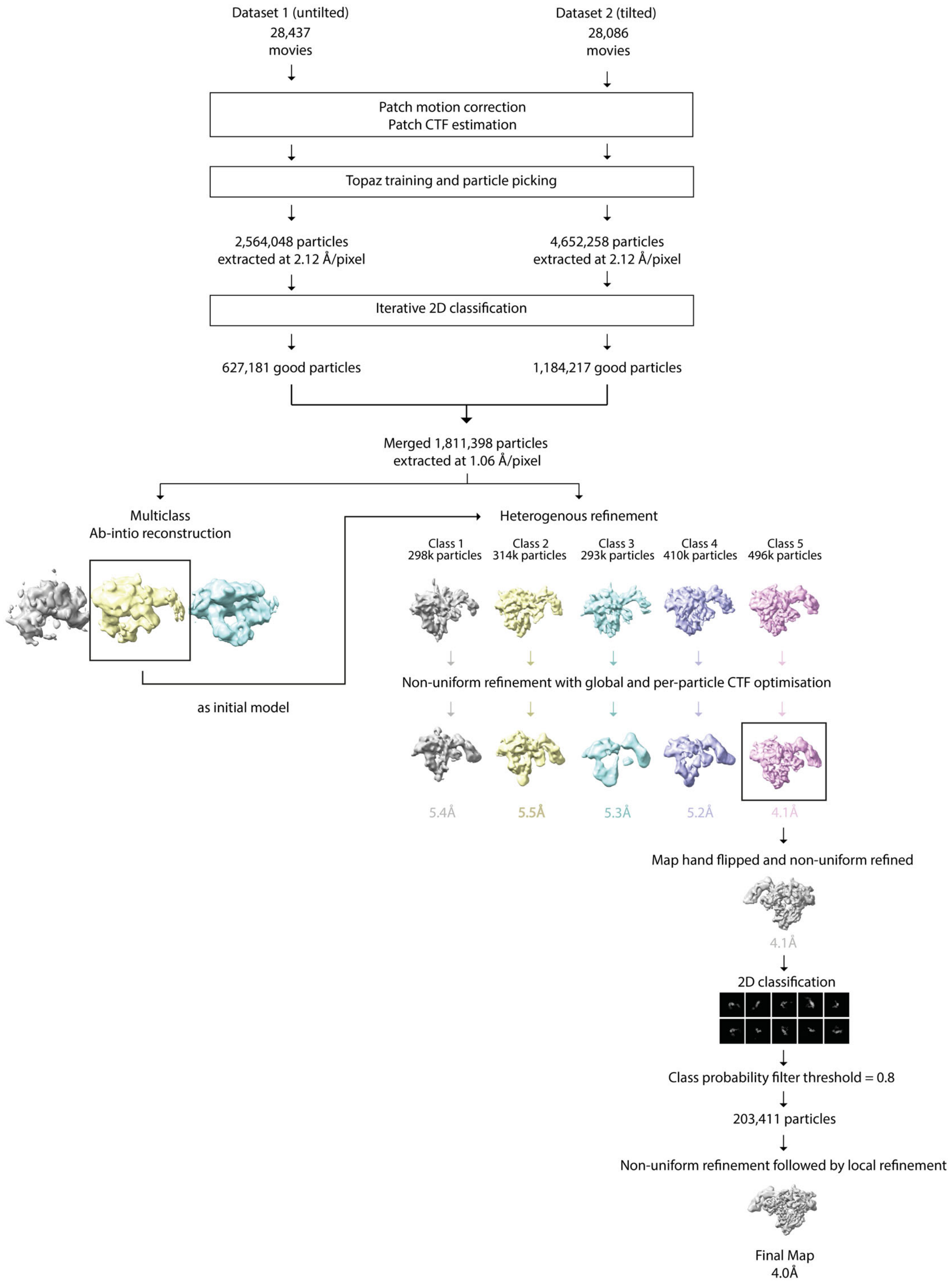

Figure EV3. CryoEM image processing workflow outlining steps performed to obtain the structure of IL-27 heterodimer complex.

All processing was performed using CryoSPARC v.3.3.1 (see Materials and Methods for details).

© 2022 The Authors

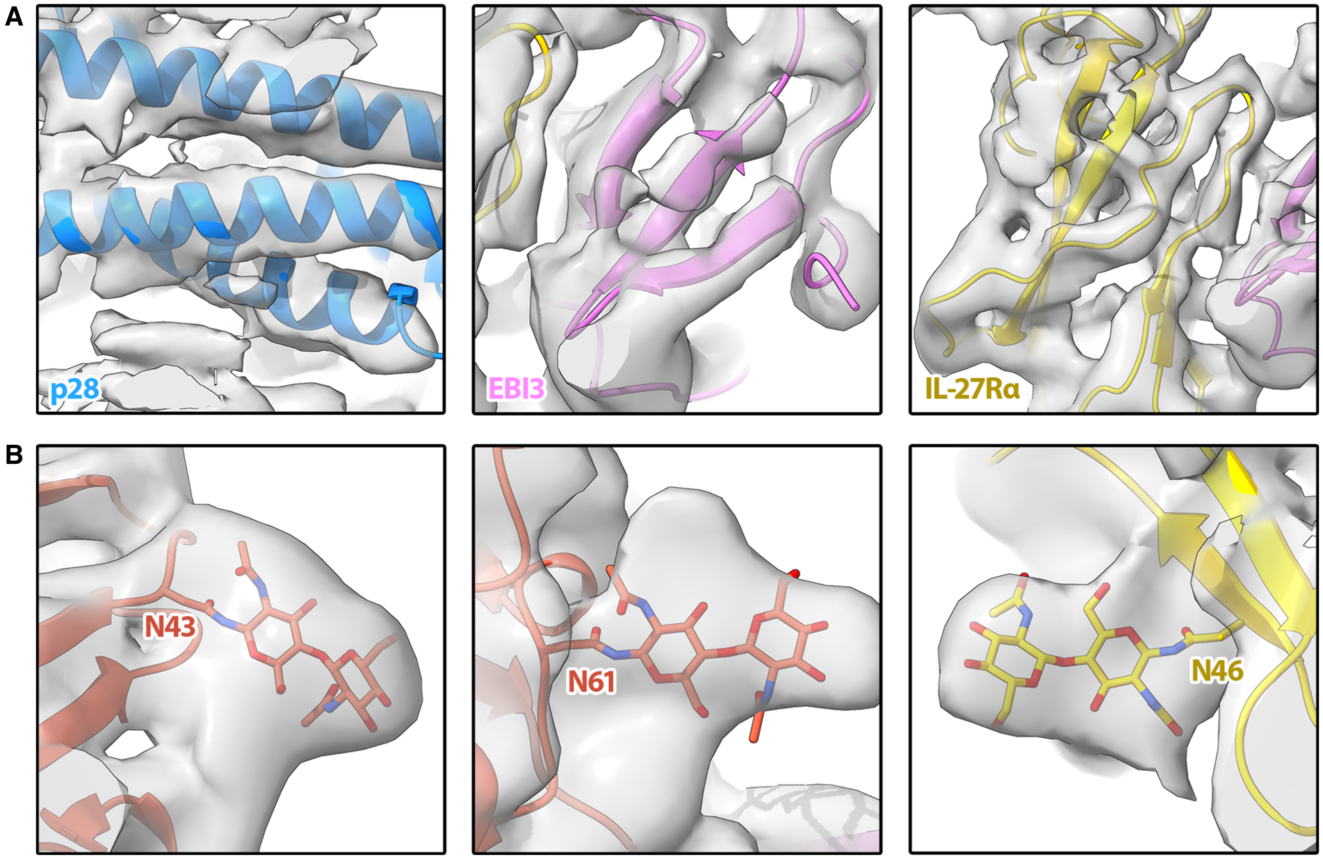

Figure EV4. Model for the IL-27 receptor signaling complex overlaid with the cryoEM map.

A Map/model overlays for density corresponding to p28 (left panel), EBI3 (middle panel), and IL-27Rα (right panel).

B Representative glycan density in the map corresponding to known glycosylation sites on GP130 (left and middle panels) and IL-27Rα (right panel).

 © 2022 The Authors

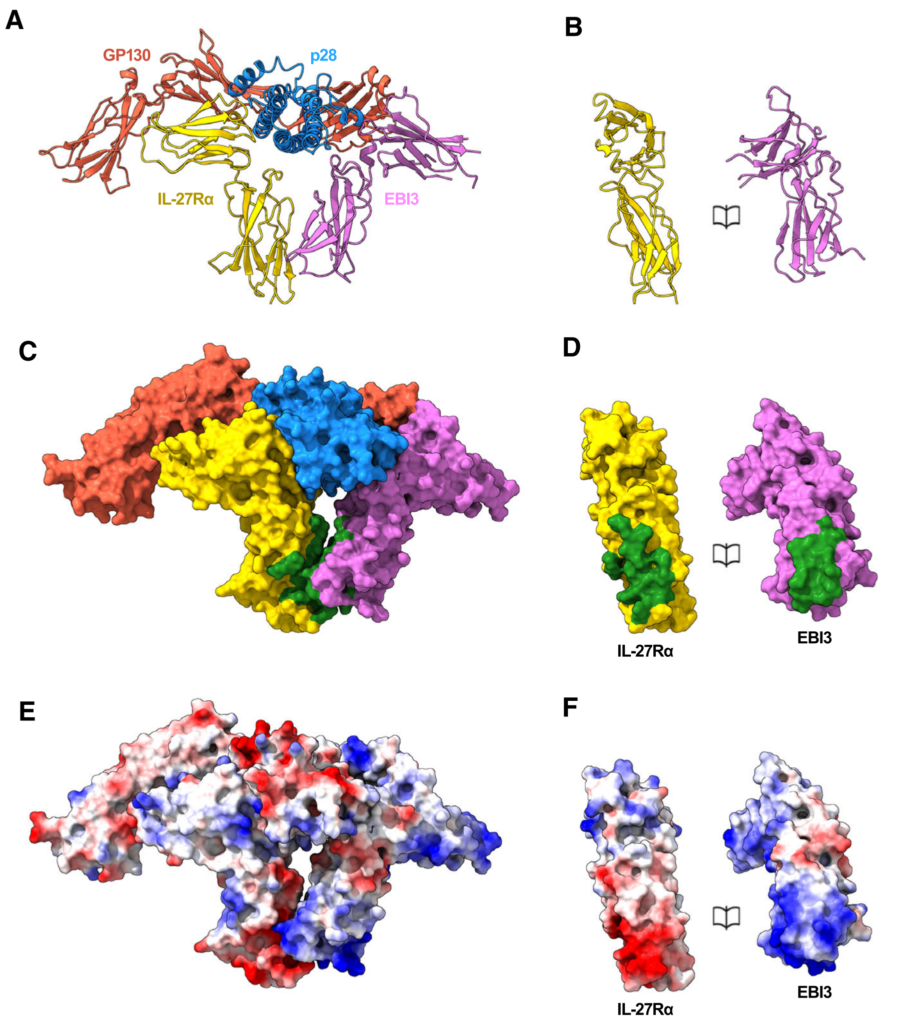

Figure EV5. EBI3:IL-27Rα interface.

A–F Ribbon representation of the IL-27 receptor recognition complex (A) and book representation of the EBI3:IL-27Rα interface (B). Individual proteins colored: EBI3 (purple), IL-27Rα (yellow), p28 (blue), GP130 (red). Surface representation of the complex (C) and book representation of the interface (D). Proteins colored as in (A) with EBI3: IL-27Rα interface residues in green. Coulombic electrostatic potential ranging from −10 (red) to 10 (blue) kcal/(mole) calculated from the models in A (E) and corresponding book representation of the interface (F).

© 2022 The Authors
