# Supplementary material for: Structural insights into the assembly and activation of the IL‐27 signaling complex
Source: EMBO Rep. 2022 Aug 3;23(10):e55450. doi: 10.15252/embr.202255450 (PMC9535766; doi:10.15252/embr.202255450)
Supplement: Supplementary file 2 — Table EV1 [file EMBR-23-e55450-s003.pdf]

CryoEM data collection and model validation statistics

	IL-27:IL-27Rα(D1-2):GP130(D1-3)	
**Data Collection and processing**	Dataset1	Dataset2
**Microscope**	Titan Krios	Titan Krios
**Camera**	K3	K3
**Magnification**	81,000x	81,000x
**Voltage**	300	300
**Electron exposure (e-/Å^2)**	50	50
**Defocus range (µm)**	-0.5 to -2.25	-1.5 to -2.7
**Pixel Size (Å/pixel)**	1.06	1.06
**Symmetry imposed**	C1	C1
**Initial micrographs**	28,437 (0° tilt)	21,474 (30° tilt)<br>6,12 (35° tilt)
**Final micrographs**	17,228	18,838
**Particles from 2D classification**	627,181	1,184,217
**Final particle images**	203,411 (Merged)	
**Global map resolution (Å)**	4.0	
**FSC threshold**	0.143	
**Map sharpening B factor (Å^2)**	-100	
**Model statistics**		
**Non hydrogen atoms**	7,040	
**Protein residues**	856	
**Ligands**	0	
**B factors**	192.42	
**R.M.S deviations**		
**Bond lengths**	0.004	
**Bond angles**	0.755	
**MolProbity score**	1.79	
**Clashscore**	8.06	
**Poor rotamers (%)**	0.65	
**Ramachandran plot**		
**Favoured (%)**	94.88	
**Allowed (%)**	5.12	
**Outliers (%)**	0	
