# Supplementary material for: Structural insights into the assembly and activation of the IL‐27 signaling complex
Source: EMBO Rep. 2022 Aug 3;23(10):e55450. doi: 10.15252/embr.202255450 (PMC9535766; doi:10.15252/embr.202255450)
Supplement: Supplementary file 4 [file EMBR-23-e55450-s004.pdf]

EMBO reports

Structural insights into the assembly and activation of the IL-27 signalling complex

Yibo Jin, Paul Fyfe, Scott Gardner, Stephan Wilmes, Doryen Bubeck, and Ignacio Moraga
DOI: 10.15252/embr.202255450

Corresponding author(s): Doryen Bubeck (d.bubeck@imperial.ac.uk) , Ignacio Moraga (i.moragagonzalez@dundee.ac.uk)

Review Timeline:

Submission Date:	20th May 22
Editorial Decision:	7th Jun 22
Revision Received:	16th Jun 22
Editorial Decision:	7th Jul 22
Revision Received:	11th Jul 22
Accepted:	11th Jul 22

Editor: Achim Breiling

Transaction Report:

(Note: With the exception of the correction of typographical or spelling errors that could be a source of ambiguity, letters and reports are not edited. Depending on transfer agreements, referee reports obtained elsewhere may or may not be included in this compilation. Referee reports are anonymous unless the Referee chooses to sign their reports.)

1st Editorial Decision 7th Jun 2022

Dear Dr. Bubeck

Thank you for the transfer of your research manuscript to EMBO reports. I have now received the reports from the three referees that were asked to evaluate your study, which can be found at the end of this email.

As you will see, the referees think that these findings are of interest. All referees have several comments, concerns, and suggestions to improve the study, which I ask you to address in a revised and expanded manuscript.

Given the constructive referee comments, we would like to invite you to revise your manuscript with the understanding that all referee concerns must be addressed in the revised manuscript and in a detailed point-by-point response. Acceptance of your manuscript will depend on a positive outcome of a second round of review. It is EMBO reports policy to allow a single round of revision only and acceptance of the manuscript will therefore depend on the completeness of your responses included in the next, final version of the manuscript.

Revised manuscripts should be submitted within three months of a request for revision. Please contact me to discuss the revision (also by video chat) if you have questions or comments regarding the revision, or should you need additional time.

When submitting your revised manuscript, please also carefully review the instructions that follow below.

PLEASE NOTE THAT upon resubmission revised manuscripts are subjected to an initial quality control prior to exposition to re-review. Upon failure in the initial quality control, the manuscripts are sent back to the authors, which may lead to delays. Frequent reasons for such a failure are the lack of the data availability section (please see below) and the presence of statistics based on n=2 (the authors are then asked to present scatter plots or provide more data points).

When submitting your revised manuscript, we will require:

1) a .docx formatted version of the final manuscript text (including legends for main figures, EV figures and tables), but without the figures included. Figure legends should be compiled at the end of the manuscript text.

2) individual production quality figure files as .eps, .tif, .jpg (one file per figure), of main figures and EV figures. Please upload these as separate, individual files upon re-submission.

The Expanded View format, which will be displayed in the main HTML of the paper in a collapsible format, has replaced the Supplementary information. You can submit up to 5 images as Expanded View. Please follow the nomenclature Figure EV1, Figure EV2 etc. The figure legend for these should be included in the main manuscript document file in a section called Expanded View Figure Legends after the main Figure Legends section. Additional Supplementary material should be supplied as a single pdf file labeled Appendix. The Appendix should have page numbers and needs to include a table of content on the first page (with page numbers) and legends for all content. Please follow the nomenclature Appendix Figure Sx, Appendix Table Sx etc. throughout the text, and also label the figures and tables according to this nomenclature.

For more details, please refer to our guide to authors:
http://www.embopress.org/page/journal/14693178/authorguide#manuscriptpreparation

Please consult our guide for figure preparation:
http://wol-prod-cdn.literatumonline.com/pb-assets/embo-site/EMBOPress_Figure_Guidelines_061115-1561436025777.pdf

See also the guidelines for figure legend preparation:
https://www.embopress.org/page/journal/14693178/authorguide#figureformat

3) a complete author checklist, which you can download from our author guidelines (https://www.embopress.org/page/journal/14693178/authorguide). Please insert page numbers in the checklist to indicate where the requested information can be found in the manuscript. The completed author checklist will also be part of the RPF.

Please also follow our guidelines for the use of living organisms, and the respective reporting guidelines:
http://www.embopress.org/page/journal/14693178/authorguide#livingorganisms

4) that primary datasets produced in this study (e.g. RNA-seq, ChIP-seq, structural and array data) are deposited in an appropriate public database. If no primary datasets have been deposited, please also state this in a dedicated section (e.g. 'No primary datasets have been generated and deposited'), see below.

See also: http://embor.embopress.org/authorguide#datadeposition

Please remember to provide a reviewer password if the datasets are not yet public.

The accession numbers and database should be listed in a formal "Data Availability" section (placed after Materials & Methods) that follows the model below. This is now mandatory (like the COI statement). Please note that the Data Availability Section is restricted to new primary data that are part of this study. This section is mandatory. As indicated above, if no primary datasets have been deposited, please state this in this section

Data availability

The datasets produced in this study are available in the following databases:

- RNA-Seq data: Gene Expression Omnibus GSE46843 (https://www.ncbi.nlm.nih.gov/geo/query/acc.cgi?acc=GSE46843)
- [data type]: [name of the resource] [accession number/identifier/doi] ([URL or identifiers.org/DATABASE:ACCESSION])

*** Note - All links should resolve to a page where the data can be accessed. ***

Moreover, I have these editorial requests:

6) We strongly encourage the publication of original source data with the aim of making primary data more accessible and transparent to the reader. The source data will be published in a separate source data file online along with the accepted manuscript and will be linked to the relevant figure. If you would like to use this opportunity, please submit the source data (for example scans of entire gels or blots, data points of graphs in an excel sheet, additional images, etc.) of your key experiments together with the revised manuscript. If you want to provide source data, please include size markers for scans of entire gels, label the scans with figure and panel number, and send one PDF file per figure.

7) Our journal encourages inclusion of *data citations in the reference list* to directly cite datasets that were re-used and obtained from public databases. Data citations in the article text are distinct from normal bibliographical citations and should directly link to the database records from which the data can be accessed. In the main text, data citations are formatted as follows: "Data ref: Smith et al, 2001" or "Data ref: NCBI Sequence Read Archive PRJNA342805, 2017". In the Reference list, data citations must be labeled with "[DATASET]". A data reference must provide the database name, accession number/identifiers and a resolvable link to the landing page from which the data can be accessed at the end of the reference. Further instructions are available at: http://www.embopress.org/page/journal/14693178/authorguide#referencesformat

8) Regarding data quantification and statistics, please make sure that the number "n" for how many independent experiments were performed, their nature (biological versus technical replicates), the bars and error bars (e.g. SEM, SD) and the test used to calculate p-values is indicated in the respective figure legends. Please also check that all the p-values are explained in the legend, and that these fit to those shown in the figure. Please provide statistical testing where applicable. Please avoid the phrase 'independent experiment', but clearly state if these were biological or technical replicates. See also: http://www.embopress.org/page/journal/14693178/authorguide#statisticalanalysis

9) Please also note our reference format:
http://www.embopress.org/page/journal/14693178/authorguide#referencesformat

10) For microscopic images, please add scale bars of similar style and thickness to all the microscopic images, using clearly visible black or white bars (depending on the background). Please place these in the lower right corner of the images. Please do not write on or near the bars in the image but define the size in the respective figure legend.

11) We updated our journal's competing interests policy in January 2022 and request authors to consider both actual and perceived competing interests. Please review the policy https://www.embopress.org/competing-interests and update your competing interests if necessary. Please name this section 'Disclosure and Competing Interests Statement' and put it after the Acknowledgements section.

12) Please add up to five keywords to the title page.

13) Please cite and discuss the related study published in eLife (PMID: 35579417)

I look forward to seeing a revised version of your manuscript when it is ready. Please let me know if you have questions or comments regarding the revision.

Please use this link to submit your revision: https://embor.msubmit.net/cgi-bin/main.plex

Yours sincerely,

Achim Breiling

Senior Editor
EMBO Reports

Referee #1:

Interleukin 27 (IL-27) is a heterodimeric cytokine consisting of the subunits p28 and Epstein-Barr virus-induced 3 (EBi3). Signal transduction is mediated by a receptor assembled from the shared glycoprotein 130 (gp130) and the cytokine-specific receptor chain IL-27Rα. While it can be grouped with other cytokines using gp130, in its heterodimeric nature, IL-27 resembles IL-12 and IL-23. IL-27 is mainly produced by monocytes, macrophages, and dendritic cells, and its targets include T helper cells and T regulatory cells, resulting in immune down-modulation during inflammation. Although significant insights into the ligand-receptor interactions have been gained for IL-6-type cytokines, IL-12, and IL-23 in recent years, the principles of how the gp130/IL-27Rα receptor recognizes IL-27 remain to be elucidated.

Jin et al. present the cryo-EM structure of mouse IL-27 (single-chain variant) in complex with the ligand-binding ectodomains of murine gp130 (D1-D3) and murine IL-27Rα (D1-D2) at 4.0 Å resolution. The authors describe the architecture of the IL-27 complex as distinct from those of IL-12 and IL-6, with the cytokine bridging the two subunits of the receptor. The EBi3 component of the cytokine appears to fulfill an outstanding role in the assembly of the complex not only by contacting D1 of gp130 but also through electrostatic interactions with a positively charged patch on IL-27Rα. The structural study is complemented by SPR measurements based on which Jin et al. deduce the importance of the two IL-27 subunits and the succession of events during assembly of the ligand-receptor complex: gp130 is proposed to be recruited to site 3 of IL-27, after the cytokine engages IL-27Rα in a high-affinity binding event via site 2 and electrostatic interactions with EBi3. The importance of EBi3 for achieving the full signaling response in CD8 T cells is confirmed by phospho-flow analysis of STAT1 and STAT3. Finally, the authors compare the architecture of the IL-27-receptor complex with those of IL-23 and IL-6 and speculate on the structural basis and consequences of chain sharing between different cytokines.

Jin et al. use cryo-EM to show how IL-27 engages its receptor consisting of gp130 and IL-27Rα. The architecture of the ligand-receptor complex is different from complexes of similar heterodimeric cytokines IL-12 and IL-23. In addition, the authors provide insights into the affinities and probable assembly mechanism of this medically relevant cytokine-receptor system. In the referee's view, the major weaknesses of this work by Jin et al. are the limited resolution of the cryo-EM reconstruction and the fact that the same structure, however of the medical most relevant human complex, has recently been published in eLife by another group at significantly higher resolution (3.47 Å) (Caveney et al., eLife, published on May 17, 2022). In addition, the ligand-receptor interaction analyses are of low quality, which need to be carefully revisited.

As far as the low resolution of the cryo-EM reconstruction by Jin et al. is concerned, a structural study describing a protein-protein complex nowadays should include the reliable characterization of side-chain interactions in the interfaces. But this very characterization appears to be hampered by the low resolution: e.g., see first main paragraph on p.6: "These residues could be involved in a series of salt bridges.", or p.5: "... (EBI3:D205) is nearby an arginine on p28 (p28:R217) and may be important for maintaining the interface ...". But D205 appears to be too far away from R217 in Fig. 2b to form any interaction. Additional major and minor issues are summarized below:

Major points:

1) Since the overall resolution is limited to 4.0 Å, with the local resolution varying from 3.5 Å to 14.1 Å, the authors should include corresponding map regions for the interfaces and modeled side chains that are discussed and shown, e.g., in Fig. 2. This would allow the readers to assess the reliability of the structural analysis and interpretation of sidechain contacts.

2) How is it possible to model the glycans on gp130 at such low local resolution?

3) The authors should mention the paper by Caveney et al. (eLife) at the end of their discussion and reference it.

4) SPR analyses: due to the slow dissociation rates, it would be helpful to provide longer dissociation traces to distinguish between signal and drift.

5) The authors should provide a table summarizing the thermodynamically and kinetically derived KD values. It is presently not clear to the reader whether the KD data are derived from the association and dissociation kinetics or from the equilibrium resonant units.

6) Figure 3: The quality of this figure needs to be improved. Units are missing. The rate constants kon and koff should be italics and non-capitalized. Please carefully check wrong or misleading values. Fig. 3a: Does 2.90-10 mean 2.90-10 or 2.90 10-10? Please provide errors or confidence intervals in a summary table.

Minor points:

1) p.4: "a reported" is duplicated

2) Fig. 2 and Suppl. Fig. 4: The authors should use a darker yellow (or different color) for the labels of IL-27Rα.

3) p.5, bottom, "... aromatic residues ... (... P153, P152 ...) ...": prolines are not aromatic!

4) The authors might want to mention that EBi3 is structurally similar to soluble cytokine receptors.

5) Suppl. Fig. 4b+d: The interface on both proteins should be outlined precisely in these open-book representations.

6) Fig. 2d: Salt bridges between Asp / Glu residues and arginines / lysines should be indicated by dotted lines (and distances).

7) Fig. 2d: The labels for R169 and K192 appear to have been mixed up.

8) Fig. 2: Since the subpanels are labeled, it is not necessary to have colored boxes.

9) Fig. 3: Please include the units for the rate constants and the equilibrium dissociation constants, put the units in the labels on the x axes in parentheses, and use lowercase letters for the rate constants.

10) Fig. 3b: The cartoon should reflect the fact that D1 of gp130 contacts both p28 and EBi3.

11) p.7: replace "... at the doses tested." with "... at the concentrations tested."?

12) p.9, bottom: replace "... for fine-tune ..." with "... for fine-tuning ..."

Referee #2:

The manuscript describes a CryoEM structure of the IL27 signalling complex. The paper represents a significant amount of work and backs up the structure published recently by Garcia. I think the structure will be of great interest to the field.

My main suggestion to improve the manuscript is to include a model of the full receptor ectodomains (including the legs). This is not shown in either this manuscript or the Garcia manuscript. I modelled it using available structures of gp130 and the alphafold2 model of IL27R and the legs of the receptor juxtapose nicely at the membrane end.

There is a lot of missing information as detailed below which should be added

Minor comments:

IL27R is only introduced late in the introduction and not earlier when describing the receptor
Page 4: Alpha fold should read AlphaFold2
Page 10: centrifugation speed should be provided as xg not rpm
Page 10: the type of SEC column used to purify the complex is not specified
The SPR section in the methods needs more detail-for example flow rates and amount of immobilized ligand
Page 11: Tween concentration is v/v or w/v?
Page 12: flux of electrons is usually referred to as total dose
Page 12: 28,437 IMAGE STACKS
Page 12: 0.25um INCREMENTS
Page 13: how many particles were re-extracted from the merged data?
Page 13: it is not obvious what the probability threshold refers to
Page 22: There is no image of the model fit within the map - the risk of overinterpretation of side-chains at this resolution is high. This could be provided in supplementary. Alternatively, you could provide a correlation plot vs. residue number
Page 22: There are glycans modelled here but they are not listed as ligands in cryo-EM statistics table (with B-factors). I would like to see the support for these glycans at this resolution, especially to model two sugars.
Page 24, Figure: 2.9-10 should read 2.9E-10.
Page 24, Figure: the inset panel in panel C is not legible
Page 24, Figure: panel e and f: c / log nM should read - log10[cytokine] (nM)
Page 25: Figure: It would be helpful to point to the sites that are unoccupied (e.g. site 2 for IL-23), in a different colour/arrow. I find it hard to compare IL-6 with the others due to the orientation. Surely at least one of the components can have the same orientation in all three complexes for easier comparison.

Figure 3 legend says Kinetic charts for IL-27Rα (a) and GP130 (b) with inserts for affinity curves but there are no insets in panels A,B

Figure 3 legend: no mention of how many times the biacore was performed. And in future the association phase of the SPR experiments should be extended so that both affinity and kinetic models can be used. Especially as the kinetic fit in panel A is poor.

Figure 3C: Giving 4 significant figure accuracy to a Kd that has not even reached a plateau in the response/concentration plot is over-interpeting the data.

Discussion: There is some discussion about the bend between D2 and D3 which is good but there is a much larger bend between D4 and D5 (Xu et al JBC 2010) which it would be nice to discuss here.

Supp table: should read 6,612 initial micrographs. Missing units (Å2) in B-factors

Supp Fig 1a would benefit from residue numbers and the first glycine is not part of the TEV site

Supp Fig 2: missing units (A). I presume the 2D class averages are a subset not the totality?

Supp Fig 4: misplaced hyphen in EBI3:IL27-Ra interface.

Referee #3:

Cytokines are not only important mediators involved in basically all developmental and physiological processes, but also make attractive therapeutic targets due to the fact that several cytokines are overexpressed in inflammatory diseases. Design and characterization of compounds that are able to selectively target individual cytokines or their receptor(s) is hindered by the fact that structural information is only available for a small number of cytokines, cytokine receptors or the whole cytokine/receptor complexes.

In this manuscript, Yibo Jin and colleagues solve the structure of interleukin-27 (IL-27) in complex with the cytokine-binding parts of the two receptors gp130 and WSX-1 (termed IL-27Rα in the manuscript). The structure is solved using state-of-the-art cryo electron microscopy. In addition to this already significant achievement, the authors determine binding affinities for each of the proteins using surface plasmon resonance in order to get insight into the kinetic drivers of the IL- 27 signaling complex assembly. This is an important manuscript that will be of high interest not only to researchers working on IL-27, but also on other cytokines and related signaling proteins. The manuscript is well written and easy to follow, the methods are clearly described in sufficient detail, and the data are technically sound.

The study is reminiscent of a paper recently published in eLife by the Garcia lab (Caveney et al.), which also reports a structure for IL-27. The resolution of the two complexes is in a similar range (3.5 Angstrom in Caveney et al. vs. 4.0 Angstrom in Jin et al.). However, Caveney et al. generated their protein complex by fusing p28 to gp130, which does not reflect at all a physiological situation. Jin et al. chose to fuse p28 to EBI3, thereby increasing the stability of IL-27, what in the opinion of this reviewer is a much better approach compared to Caveney et al. Furthermore, while Caveney et al. only report the actual structure of the IL-27, Jin et al. in addition present biochemical and biophysical data on the kinetics of the complex formation, which is a great addition to the IL-27 structure. In summary, although an IL-27 structure was already published in eLife, this manuscript should definetly be published in a high ranking journal like EMBO Reports.

I have only two minor comments that the authors could address in order to increase the clarity of their manuscript, which are as follows:

• Gp130 and WSX-1 consist of six anf five extracellular domains, respectively. In the current version of the manuscript, only "The N-terminal immunoglobulin (Ig) domain of GP130" is mentioned. The authors should also mention and describe the other domains of both gp130 and WSX-1, which will make it much easier for the reader to understand which parts of the receptors are directly involved in complex formation. It should also be mentioned explicitly which domains are part of the final structure; "IL-27 in complex with cytokine binding domains of GP130 and its co-receptor IL-27Rα" is too vague. I see that this is mentioned in the results, but it makes more sense to move this to the introduction.
• The authors state in the abstract that "both receptors weakly bind the p28 component of the heterodimeric cytokine", while they state at the end of the results section that for p28 they measured "no binding to GP130 at the doses tested" - please clarify.

Dear Dr. Achim Breiling,

All editorial requests are now incorporated into this revision. Below is a point-by-point response to all reviewer comments.

Major points:

1) Since the overall resolution is limited to 4.0 Å, with the local resolution varying from 3.5 Å to 14.1 Å, the authors should include corresponding map regions for the interfaces and modeled side chains that are discussed and shown, e.g., in Fig. 2. This would allow the readers to assess the reliability of the structural analysis and interpretation of sidechain contacts.

At our reported resolution (4.0 Å), we can clearly resolve secondary structure elements consistent with this resolution. We now include map/model overlay of fits within the p28 helical bundle and β-strand separation within EBI3 in a new supplementary figure (Supplementary Figure 4; new numbering). However, at this resolution we do not observe clear side-chain density. Models were heavily restrained to AlphaFold2 predictions of IL-27Rα (domains 1-2), GP130 (domains 1-3), EBI3 and p28 as indicated in the text. While we acknowledge there may some uncertainty in the rotamer of the sidechains, the Cα carbon position is defined by the rigid-body fit of the AlphaFold2 models. Therefore, we have included them in the figures for visualization purposes and have clarified the text and figure legend to reflect this.

"Given the lack of side-chain density at this resolution, models were derived from Alphafold2 predictions (Jumper et al., 2021) refined with strong adaptive distance restraints and geometric restraints imposed (Appendix Table S1). While there may be some uncertainty in the sidechain rotamer, interface residues are defined largely by the rigid-body fit of AlphaFold2 models."

2) How is it possible to model the glycans on gp130 at such low local resolution?

We have clear density for glycans in our maps and now include an additional supplementary figure to directly show the fits (Supplementary Figure 4; new numbering). We only modelled glycans that satisfied two criteria: 1) where there was clear evidence for them in the density and 2) only at positions indicated to be glycosylated on the Uniprot database. Indeed, superposition of the human crystal structure for glycosylated human GP130 confirms the location of the modelled glycans.

3) The authors should mention the paper by Caveney et al. (eLife) at the end of their discussion and reference it.

We now refer to the Caveney et al. paper.

"Indeed, our data agrees with a recently published structure of the human IL-27 signalling complex (Caveney et al., 2022)."

4) SPR analyses: due to the slow dissociation rates, it would be helpful to provide longer dissociation traces to distinguish between signal and drift.

We now provide new SPR data (Revised Fig. 3) where we show dissociation times of 700 sec and 400 sec for IL-27 binding IL-27Rα and GP130, respectively.

5) The authors should provide a table summarizing the thermodynamically and kinetically derived KD values. It is presently not clear to the reader whether the KD data are derived from the association and dissociation kinetics or from the equilibrium resonant units.

We now provide a table summarizing the thermodynamically and kinetically derived KD values (Revised Fig.3e). IL-27 KD for IL-27Rα and GP130 were kinetically derived, while p28 KD for IL-27Rα was thermodynamically derived. This has been clearly stated in the revised Fig.3 legend.

6) Figure 3: The quality of this figure needs to be improved. Units are missing. The rate constants kon and koff should be italics and non-capitalized. Please carefully check wrong or misleading values. Fig. 3a: Does 2.90-10 mean 2.90-10 or 2.90×10-10? Please provide errors or confidence intervals in a summary table.

We have now implemented all the reviewer suggestions.

Minor points:

1) p.4: "a reported" is duplicated

This is now done.

2) Fig. 2 and Suppl. Fig. 4: The authors should use a darker yellow (or different color) for the labels of IL-27Rα.

We have changed the color of the labels for better visibility.

3) p.5, bottom, "... aromatic residues ... (... P153, P152 ...) ...": prolines are not aromatic!

This has now been changed in the text.

"Docking and minimal refinement of the EBI3 Alphafold2 model into the map show that the bend of the EBI3 elbow is formed by a cluster of aromatic residues (EBI3: Y39, F96, F159, and Y209) together with a proline (EBI3:P40), which create a hydrophobic groove recognized by p28:W93 (Figure 2b)."

“Here we find that the knob and hole pattern is encoded by cluster of proline residues (IL-27Rα: P153, P152) and an aromatic residue (W151) of IL-27Rα, which form a pocket to bind a tyrosine extending from p28 (p28:Y48)”

4) The authors might want to mention that EBi3 is structurally similar to soluble cytokine receptors.

This has now been changed in the text.

“While p28 exhibits a classical four-α helical fold, EBI3 is structurally similar to soluble cytokine receptors (Rousseau et al., 2010).”

5) Suppl. Fig. 4b+d: The interface on both proteins should be outlined precisely in these open-book representations.

We have now colored the interface residues as defined by Pisa and included this as an additional panel in Figure EV5 (new numbering) to aide in the interpretation of the electrostatics image.

6) Fig. 2d: Salt bridges between Asp / Glu residues and arginines / lysines should be indicated by dotted lines (and distances).

Given the positional uncertainty of the sidechain rotamer at this resolution, we do not feel it is appropriate to specifically define a salt bridge.

7) Fig. 2d: The labels for R169 and K192 appear to have been mixed up.

This is now fixed.

8) Fig. 2: Since the subpanels are labeled, it is not necessary to have colored boxes.

We have now removed the colored boxes from the subpanels.

9) Fig. 3: Please include the units for the rate constants and the equilibrium dissociation constants, put the units in the labels on the x axes in parentheses, and use lowercase letters for the rate constants.

This is now done.

10) Fig. 3b: The cartoon should reflect the fact that D1 of gp130 contacts both p28 and EBi3.

This is now fixed.

11) p.7: replace "... at the doses tested." with "... at the concentrations tested."?

This is now fixed.

12) p.9, bottom: replace "... for fine-tune ..." with "... for fine-tuning ..."

This is now done.

Referee #2:

The manuscript describes a CryoEM structure of the IL27 signalling complex. The paper represents a significant amount of work and backs up the structure published recently by Garcia. I think the structure will be of great interest to the field.

My main suggestion to improve the manuscript is to include a model of the full receptor ectodomains (including the legs). This is not shown in either this manuscript or the Garcia manuscript. I modelled it using available structures of gp130 and the alphafold2 model of IL27R and the legs of the receptor juxtapose nicely at the membrane end.

We have now modelled the complete signalling complex based on the AlphaFold2 predictions of the full-length receptors and include it in Figure 1c.

There is a lot of missing information as detailed below which should be added

Minor comments:

IL27R is only introduced late in the introduction and not earlier when describing the receptor.

We have now added a sentence in the first paragraph of the introduction to introduce IL27Ra earlier in the text.

"Understanding how IL-27 engages its cellular receptors, GP130 and IL-27Rα, to form a signalling complex will provide fundamental insight into immune regulation, which could facilitate the development of new therapeutics that target IL-27 responses."

Page 4: Alpha fold should read AlphaFold2

This now corrected on page 4 and all other instances in the text.

Page 10: centrifugation speed should be provided as xg not rpm

This is now fixed.

Page 10: the type of SEC column used to purify the complex is not specified

This is now fixed: ENrich SEC 650 10 x 300 column (Biorad)

The SPR section in the methods needs more detail-for example flow rates and amount of immobilized ligand

The SPR method section has now been expanded to include the details requested by the reviewer.

Page 11: Tween concentration is v/v or w/v?

This is now fixed in the text. Tween concentration is v/v.

Page 12: flux of electrons is usually referred to as total dose

This is now changed.

Page 12: 28,437 IMAGE STACKS

This is now changed.

Page 12: 0.25um INCREMENTS

This is now changed.

Page 13: how many particles were re-extracted from the merged data?

1,811,398 particles were extracted from the merged data. This information was present in the cryoEM workflow supplementary figure and has now been added to the methods section.

Page 13: it is not obvious what the probability threshold refers to

The probability threshold is defined as a value between 0 and 1 and excludes particles with smaller 2D class posterior probabilities compared with probabilities from the particles.alignments2D. It is a standard option in the Cryosparc workflow v3.3.1. We have clarified this in the text.

Page 22: There is no image of the model fit within the map - the risk of overinterpretation of side-chains at this resolution is high. This could be provided in supplementary. Alternatively, you could provide a correlation plot vs. residue number

We have now provided several map/model fits in the supplementary information and made it more clear to the reader how interaction interfaces are interpreted. See response to Reviewer 1 point 1.

Page 22: There are glycans modelled here but they are not listed as ligands in cryo-EM statistics table (with B-factors). I would like to see the support for these glycans at this resolution, especially to model two sugars.

Support for glycan modelling is now included in the Supplementary Information. See response to Reviewer 1 point 2. We have also included an additional line in the Supplementary Table 1 to include glycan B-factors (Ligand).

Page 24, Figure: 2.9-10 should read 2.9E-10.

This is now fixed.

Page 24, Figure: the inset panel in panel C is not legible

This is now fixed.

Page 24, Figure: panel e and f: c / log nM should read - log10[cytokine] (nM)

This is now corrected.

Page 25: Figure: It would be helpful to point to the sites that are unoccupied (e.g. site 2 for IL-23), in a different colour/arrow. I find it hard to compare IL-6 with the others due to the orientation. Surely at least one of the components can have the same orientation in all three complexes for easier comparison.

We have modified the Figure to include white and grey circles to indicate occupied and unoccupied sites, respectively. In the original version of the figure models were all oriented based on superposition of GP130. We have now changed the reference frame to be centered on p28 (or equivalent helical cytokine).

Figure 3 legend says Kinetic charts for IL-27Rα (a) and GP130 (b) with inserts for affinity curves but there are no insets in panels A,B

This is now fixed.

Figure 3 legend: no mention of how many times the biacore was performed. And in future the association phase of the SPR experiments should be extended so that both affinity and kinetic models can be used. Especially as the kinetic fit in panel A is poor.

This is now fixed. We have redone the SPR experiment to increase the association time to 120 secs (Revised Fig.3). The biacore experiment was done three times and this have now been clearly stated in the Fig. 3 legend.

Figure 3C: Giving 4 significant figure accuracy to a Kd that has not even reached a plateau in the response/concentration plot is over-interpeting the data.

This is now fixed.

Discussion: There is some discussion about the bend between D2 and D3 which is good but there is a much larger bend between D4 and D5 (Xu et al JBC 2010) which it would be nice to discuss here.

We have now included this in our discussion.

"Indeed, when we extend our model to include the full-length domains of IL-27Rα and GP130, the bend between D4 and D5 of GP130 (Xu et al., 2010) enables the juxtaposition of the membrane proximal domains of the two signalling receptors (Figure 1c)."

Supp table: should read 6,612 initial micrographs. Missing units (Å2) in B-factors

The Appendix Table S1 has now been modified to include these changes.

Supp Fig 1a would benefit from residue numbers and the first glycine is not part of the TEV site

Numbers have now been included in the revised Figure EV1a.

Supp Fig 2: missing units (A). I presume the 2D class averages are a subset not the totality?

The legend has now been modified to clarify that these are a subset of the 2D class averages and the units are now included in the color scale bar.

Supp Fig 4: misplaced hyphen in EBI3:IL27-Ra interface.

This is now fixed.

Referee #3:

Cytokines are not only important mediators involved in basically all developmental and physiological processes, but also make attractive therapeutic targets due to the fact that several cytokines are overexpressed in inflammatory diseases. Design and characterization of compounds that are able to selectively target individual cytokines or their receptor(s) is hindered by the fact that structural information is only available for a small number of cytokines, cytokine receptors or the whole cytokine/receptor complexes.

In this manuscript, Yibo Jin and colleagues solve the structure of interleukin-27 (IL-27) in

complex with the cytokine-binding parts of the two receptors gp130 and WSX-1 (termed IL-27Rα in the manuscript). The structure is solved using state-of-the-art cryo electron microscopy. In addition to this already significant achievement, the authors determine binding affinities for each of the proteins using surface plasmon resonance in order to get insight into the kinetic drivers of the IL- 27 signaling complex assembly. This is an important manuscript that will be of high interest not only to researchers working on IL-27, but also on other cytokines and related signaling proteins. The manuscript is well written and easy to follow, the methods are clearly described in sufficient detail, and the data are technically sound.

The study is reminiscent of a paper recently published in eLife by the Garcia lab (Caveney et al.), which also reports a structure for IL-27. The resolution of the two complexes is in a similar range (3.5 Angstrom in Caveney et al. vs. 4.0 Angstrom in Jin et al.). However, Caveney et al. generated their protein complex by fusing p28 to gp130, which does not reflect at all a physiological situation. Jin et al. chose to fuse p28 to EBI3, thereby increasing the stability of IL-27, what in the opinion of this reviewer is a much better approach compared to Caveney et al. Furthermore, while Caveney et al. only report the actual structure of the IL-27, Jin et al. in addition present biochemical and biophysical data on the kinetics of the complex formation, which is a great addition to the IL-27 structure. In summary, although an IL-27 structure was already published in eLife, this manuscript should definetly be published in a high ranking journal like EMBO Reports.

I have only two minor comments that the authors could address in order to increase the clarity of their manuscript, which are as follows:

• Gp130 and WSX-1 consist of six and five extracellular domains, respectively. In the current version of the manuscript, only "The N-terminal immunoglobulin (Ig) domain of GP130" is mentioned. The authors should also mention and describe the other domains of both gp130 and WSX-1, which will make it much easier for the reader to understand which parts of the receptors are directly involved in complex formation. It should also be mentioned explicitly which domains are part of the final structure; "IL-27 in complex with cytokine binding domains of GP130 and its co-receptor IL-27Rα" is too vague. I see that this is mentioned in the results, but it makes more sense to move this to the introduction.

We have now explicitly described which domains are part of our final structure in the introduction. We have also included an Alphafold2-derrived model for the entire extracellular domains of the complex and included in Fig. 1c as requested by Reviewer 2 to provide context.

"Here, we report a 4.0 Å resolution cryo-EM structure of IL-27 in complex with cytokine binding domains of GP130 (domains 1-3) and its co-receptor IL-27Rα (domains 1-2)."

• The authors state in the abstract that "both receptors weakly bind the p28 component of the heterodimeric cytokine ", while they state at the end of the results section that for p28 they measured "no binding to GP130 at the doses tested" - please clarify.

We have now clarified this the abstract.

“While both receptors contact the p28 component of the heterodimeric cytokine, EBI3 stabilizes the complex by binding a positively charged surface of IL-27Rα and Domain 1 of GP130.”

1st Revision - Editorial Decision 7th Jul 2022

Dear Dr. Bubeck,

Thank you for the submission of your revised manuscript to our editorial offices. I have now received the reports from the three referees that were asked to re-evaluate your study, you will find below. As you will see, the referees now support publication of the study in EMBO reports. Referees #1 and #2 have remaining points and suggestions to improve the manuscript I ask you to address in a final revised manuscript. Please also provide a final p-b-p-response addressing the remaining points of the referees.

Moreover, I have these editorial requests:

- Please choose one of these two alternative titles:
Structural insights into the assembly and activation of IL-27 signalling complexes
Structural insights into the assembly and activation of the IL-27 signalling complex

- We plan to publish your manuscript in the Report format. For a Scientific Report we require that results and discussion sections are combined in a single chapter called "Results & Discussion". Please do this for your manuscript. For more details please refer to our guide to authors: http://www.embopress.org/page/journal/14693178/authorguide#researcharticleguide

- Please order the manuscript sections like this, using these names:
Title page - Abstract - Key Words - Introduction - Results & Discussion - Materials and Methods - Data availability section - Acknowledgements - Author contributions - Disclosure and Competing Interests Statement - References - Figure legends - Expanded View Figure legends

- It seems presently panel 3E is called out before 3C. Moreover, there are mostly no separate callouts for the EV figure panels. Please check and make sure that all figure panels (main and EV figures) are called out separately and sequentially.

- Please label the figure panels uniformly with capital letters (A, B, C, ...).

- Please add scale bars of similar style and thickness to all the microscopic images (main and EV figures), using clearly visible black or white bars (depending on the background). Please place these in the lower right corner of the images. Please do not write on or near the bars in the image but define the size in the respective figure legend.

- Please upload the Appendix table as Table EV1 (so we do not need an Appendix). Please upload the table as separate file and add a legend for it after the EV figure legends. Please also give it a title.

- Please check that in the final Data availability section direct links to the datasets are provided and that no referee tokens are shown anymore. Please also make sure that the datasets are public latest upon publication of the study.

- Finally, please find attached a word file of the manuscript text (provided by our publisher) with changes we ask you to include in your final manuscript text, and some queries, we ask you to address. Please provide your final manuscript file with track changes, in order that we can see any modifications done.

In addition, I would need from you:
- a short, two-sentence summary of the manuscript (around 35 words).
- three to four short bullet points highlighting the key findings of your study
- a schematic summary figure (in jpeg or tiff format with the exact width of 550 pixels and a height of not more than 400 pixels) that can be used as a visual synopsis on our website.

I look forward to seeing the final revised version of your manuscript when it is ready. Please let me know if you have questions regarding the revision.

Best,

Achim Breiling
Senior Editor
EMBO Reports

Referee #1:

The critical concerns by the reviewers have been addressed accordingly, thereby strengthening to the conclusions. Overall, the quality of manuscript improved. However, the structure of the IL-27 receptor recognition complex is still at limited resolution and

the final model largely biased using AlphaFold2. This should be clearly stated in the abstract. In addition, the abstract leaves the impression that full-length ectodomains are used. It should be stated that only the ectodomains D1-D3 of D1-D5 have been examined. It is further misleading that the authors refer to the full-length domains of IL-27Ralpha and GP130 (see Figure 1c). It should read "soluble full-length ectodomains".

Referee #2:

The revised manuscript is now acceptable for publication with one minor exception. The model for the full ectodomain receptor in figure 1 is not similar to the one I have generated here. This may be (1) because of slight differences between the Garcia structure (PDB 7U7N) and the structure determined by these authors (which is on hold in the PDB therefore I could not use it for modelling. Or (2) it may be due to incorrect modelling by the authors. If it is (1) then the authors should mention any significant structural differences in the text. If it is (2) then the authors can model the complex using the following method:

Take their structure. Import the alphafold model of the IL27R and align it to theirs. Import the PDB gp130 structure (ID 3L5H) and align to the author's gp130 fragment.

The main difference is that when I do it the legs of the 2 receptor chains contact at the membrane proximal end and are not separated like in the author's figure 1C.

Referee #3:

I had only minor comments on the previous version of the manuscript, and these have been addressed in full by the authors. I recommend to publish the manuscript in EMBO Reports and congratulate the authors!

Dear Dr. Achim Breiling,

We have now revised the manuscript addressing all final editorial and reviewer responses.

- Please choose one of these two alternative titles:
Structural insights into the assembly and activation of IL-27 signalling complexes
Structural insights into the assembly and activation of the IL-27 signalling complex

We have changed the title to *"Structural insights into the assembly and activation of the IL-27 signalling complex"*.

- We plan to publish your manuscript in the Report format. For a Scientific Report we require that results and discussion sections are combined in a single chapter called "Results & Discussion". Please do this for your manuscript. For more details please refer to our guide to authors: http://www.embopress.org/page/journal/14693178/authorguide#researcharticleguide

The manuscript is now revised to have a Results & Discussion section.

- Please order the manuscript sections like this, using these names:
Title page - Abstract - Key Words - Introduction - Results & Discussion - Materials and Methods - Data availability section - Acknowledgements - Author contributions - Disclosure and Competing Interests Statement - References - Figure legends - Expanded View Figure legends

This is now done.

- It seems presently panel 3E is called out before 3C. Moreover, there are mostly no separate callouts for the EV figure panels. Please check and make sure that all figure panels (main and EV figures) are called out separately and sequentially.

We have adjusted call outs in the text.

- Please label the figure panels uniformly with capital letters (A, B, C, ...).

This is now done.

- Please add scale bars of similar style and thickness to all the microscopic images (main and EV figures), using clearly visible black or white bars (depending on the background). Please place these in the lower right corner of the images. Please do not write on or near the bars in the image but define the size in the respective figure legend.

We have now modified the scale bars for consistency in the microscopy images presented in Figure EV2 and removed the number value from these panels. We would prefer to keep the main text Figure 1 with the distance values as is because it gives information on the dimensions of the complex.

- Please upload the Appendix table as Table EV1 (so we do not need an Appendix). Please upload the table as separate file and add a legend for it after the EV figure legends. Please also give it a title.

We now refer to the table as Table EV1 in the text. The table does not require a legend. We have now given it a title and added it after the EV figure legends.

- Please check that in the final Data availability section direct links to the datasets are provided and that no referee tokens are shown anymore. Please also make sure that the datasets are public latest upon publication of the study.

I have now removed the referee passwords from the Data availability section and will update the direct links to the entries at the proof stages when the data is released for publication.

- Finally, please find attached a word file of the manuscript text (provided by our publisher) with changes we ask you to include in your final manuscript text, and some queries, we ask you to address. Please provide your final manuscript file with track changes, in order that we can see any modifications done.

The manuscript edits are done in track changes.

In addition, I would need from you:
- a short, two-sentence summary of the manuscript (around 35 words).

Structure of the IL-27 signalling complex provides a mechanistic blueprint to fine-tune IL-27 immuno-modulatory activities.

- three to four short bullet points highlighting the key findings of your study

- IL-27 is a heterodimeric cytokine in which EBI3 engages p28 at site 1
- IL-27Rα binds site 2 of p28 and is stabilized by electrostatic interactions with EBI3
- Domain 1 of GP130 binds site 3 in a low affinity interaction

- a schematic summary figure (in jpeg or tiff format with the exact width of 550 pixels and a height of not more than 400 pixels) that can be used as a visual synopsis on our website.

We have generated the graphic and include it in the resubmission.

Referee #1:

The critical concerns by the reviewers have been addressed accordingly, thereby strengthening to the conclusions. Overall, the quality of manuscript improved. However, the structure of the IL-27 receptor recognition complex is still at limited resolution and the final model largely biased using AlphaFold2. This should be clearly stated in the abstract.

We now explicitly mention the use of AlphaFold2 in the abstract.

"Here, we used cryo electron microscopy (cryoEM) and AlphaFold2 modelling to solve the structure of the IL-27 receptor recognition complex."

In addition, the abstract leaves the impression that full-length ectodomains are used. It should be stated that only the ectodomains D1-D3 of D1-D5 have been examined.

We have now explicitly stated the domains used in our cryoEM studies in the abstract.

"Our data show how IL-27 serves as a bridge connecting IL-27Rα (domains 1-2) with GP130 (domains 1-3) to initiate signalling."

It is further misleading that the authors refer to the full-length domains of IL-27Ralpha and GP130 (see Figure 1c). It should read "soluble full-length ectodomains".

We have removed panel C in this figure and have removed this line from the text accordingly.

Referee #2:

The revised manuscript is now acceptable for publication with one minor exception. The model for the full ectodomain receptor in figure 1 is not similar to the one I have generated here. This may be (1) because of slight differences between the Garcia structure (PDB 7U7N) and the structure determined by these authors (which is on hold in the PDB therefore I could not use it for modelling. Or (2) it may be due to incorrect modelling by the authors. If it is (1) then the authors should mention any significant structural differences in the text. If it is (2) then the authors can model the complex using the following method:

Take their structure. Import the alphafold model of the IL27R and align it to theirs. Import the PDB gp130 structure (ID 3L5H) and align to the author's gp130 fragment.

The main difference is that when I do it the legs of the 2 receptor chains contact at the membrane proximal end and are not separated like in the author's figure 1C.

Superposition of the Garcia structure (PDB 7U7N) with our model are largely in agreement, with subtle differences in the rotation of the receptors relative to the core p28 cytokine. This is now clarified in the text.

"Our data agrees with a recently published structure of the human IL-27 signalling complex (Caveney et al., 2022), with slight differences in rotation of the receptors relative to the helical cytokine"

While these slight rotations do not change the identity of the interaction interface residues, they do lead to differences in the trajectories of full-length proteins in the extended AlphaFold models as asked for by the reviewer. Given the modest resolution of our map, we feel it is appropriate to remove the panel C and tone down our discussion of the distance between cytoplasmic domains.

Referee #3:

I had only minor comments on the previous version of the manuscript, and these have been addressed in full by the authors. I recommend to publish the manuscript in EMBO Reports and congratulate the authors!

Dr. Doryen Bubeck
Imperial College London
Department of Life Sciences
Imperial College
London sw72az
United Kingdom

Dear Dr. Bubeck,

I am very pleased to accept your manuscript for publication in the next available issue of EMBO reports. Thank you for your contribution to our journal.

At the end of this email I include important information about how to proceed. Please ensure that you take the time to read the information and complete and return the necessary forms to allow us to publish your manuscript as quickly as possible.

As part of the EMBO publication's Transparent Editorial Process, EMBO reports publishes online a Review Process File to accompany accepted manuscripts. As you are aware, this File will be published in conjunction with your paper and will include the referee reports, your point-by-point response and all pertinent correspondence relating to the manuscript.

If you do NOT want this File to be published, please inform the editorial office within 2 days, if you have not done so already, otherwise the File will be published by default [contact: emboreports@embo.org]. If you do opt out, the Review Process File link will point to the following statement: "No Review Process File is available with this article, as the authors have chosen not to make the review process public in this case." Please note that the author checklist will still be published even if you opt out of the transparent process.

Thank you again for your contribution to EMBO reports and congratulations on a successful publication. Please consider us again in the future for your most exciting work.

Yours sincerely,

Achim Breiling
Editor
EMBO Reports

****

THINGS TO DO NOW:

Once your article has been received by Wiley for production, the corresponding author will receive an email from Wiley's Author Services system which will ask them to log in and will present them with the appropriate license for completion.

You will receive proofs by e-mail approximately 2-3 weeks after all relevant files have been sent to our Production Office; you should return your corrections within 2 days of receiving the proofs.

Please inform us if there is likely to be any difficulty in reaching you at the above address at that time. Failure to meet our deadlines may result in a delay of publication, or publication without your corrections.

All further communications concerning your paper should quote reference number EMBOR-2022-55450V3 and be addressed to emboreports@wiley.com.

Should you be planning a Press Release on your article, please get in contact with emboreports@wiley.com as early as possible, in order to coordinate publication and release dates.

EMBO Press Author Checklist

Corresponding Author Name: Doryen Bubeck and Inacio Moraga	
Journal Submitted to: EMBO Reports	
Manuscript Number: EMBOR-2022-55450-T	

USEFUL LINKS FOR COMPLETING THIS FORM
The EMBO Journal - Author Guidelines
EMBO Reports - Author Guidelines
Molecular Systems Biology - Author Guidelines
EMBO Molecular Medicine - Author Guidelines

Reporting Checklist for Life Science Articles (updated January 2022)
This checklist is adapted from Materials Design Analysis Reporting (MDAR) Checklist for Authors. MDAR establishes a minimum set of requirements in transparent reporting in the life sciences (see Statement of Task: 10.31222/osf.io/9sm4x). Please follow the journal's guidelines in preparing your manuscript.
Please note that a copy of this checklist will be published alongside your article.

Abridged guidelines for figures
1. Data
The data shown in figures should satisfy the following conditions:
- the data were obtained and processed according to the field's best practice and are presented to reflect the results of the experiments in an accurate and unbiased manner.
- ideally, figure panels should include only measurements that are directly comparable to each other and obtained with the same assay.
- plots include clearly labeled error bars for independent experiments and sample sizes. Unless justified, error bars should not be shown for technical replicates.
- if n<5, the individual data points from each experiment should be plotted. Any statistical test employed should be justified.
- Source Data should be included to report the data underlying figures according to the guidelines set out in the authorship guidelines on Data Presentation.

2. Captions
Each figure caption should contain the following information, for each panel where they are relevant:
- a specification of the experimental system investigated (eg cell line, species name).
- the assay(s) and method(s) used to carry out the reported observations and measurements.
- an explicit mention of the biological and chemical entity(ies) that are being measured.
- an explicit mention of the biological and chemical entity(ies) that are altered/varied/perturbed in a controlled manner.
- the exact sample size (n) for each experimental group/condition, given as a number, not a range;
- a description of the sample collection allowing the reader to understand whether the samples represent technical or biological replicates (including how many animals, litters, cultures, etc.).
- a statement of how many times the experiment shown was independently replicated in the laboratory.
- definitions of statistical methods and measures:
  - common tests, such as t-test (please specify whether paired vs. unpaired), simple χ2 tests, Wilcoxon and Mann-Whitney tests, can be unambiguously identified by name only, but more complex techniques should be described in the methods section;
  - are tests one-sided or two-sided?
  - are there adjustments for multiple comparisons?
  - exact statistical test results, e.g., P values = x but not P values < x;
  - definition of 'center values' as median or average;
  - definition of error bars as s.d. or s.e.m.

Please complete ALL of the questions below.
Select "Not Applicable" only when the requested information is not relevant for your study.

Materials

Newly Created Materials	Information included in the manuscript?	In which section is the information available? (Reagents and Tools Table, Materials and Methods, Figures, Data Availability Section)
New materials and reagents need to be available; do any restrictions apply?	Not Applicable	There are no restrictions on new material generated and are available upon request.

Antibodies	Information included in the manuscript?	In which section is the information available? (Reagents and Tools Table, Materials and Methods, Figures, Data Availability Section)
For **antibodies** provide the following information:<br>- Commercial antibodies: RRID (if possible) or supplier name, catalogue number and or/clone number<br>- Non-commercial: RRID or citation	Not Applicable	

DNA and RNA sequences	Information included in the manuscript?	In which section is the information available? (Reagents and Tools Table, Materials and Methods, Figures, Data Availability Section)
**Short novel DNA or RNA including primers, probes**: provide the sequences.	Not Applicable	

Cell materials	Information included in the manuscript?	In which section is the information available? (Reagents and Tools Table, Materials and Methods, Figures, Data Availability Section)
**Cell lines:** Provide species information, strain. Provide accession number in repository **OR** supplier name, catalog number, clone number, and/**OR** RRID.	Yes	Materials and Methods
**Primary cultures:** Provide species, strain, sex of origin, genetic modification status.	Yes	Materials and Methods
Report if the cell lines were recently **authenticated** (e.g., by STR profiling) and tested for mycoplasma contamination.	Not Applicable	

Experimental animals	Information included in the manuscript?	In which section is the information available? (Reagents and Tools Table, Materials and Methods, Figures, Data Availability Section)
**Laboratory animals or Model organisms:** Provide species, strain, sex, age, genetic modification status. Provide accession number in repository **OR** supplier name, catalog number, clone number, **OR** RRID.	Not Applicable	
**Animal observed in or captured from the field:** Provide species, sex, and age where possible.	Not Applicable	
Please detail **housing and husbandry conditions**.	Not Applicable	

Plants and microbes	Information included in the manuscript?	In which section is the information available? (Reagents and Tools Table, Materials and Methods, Figures, Data Availability Section)
**Plants:** provide species and strain, ecotype and cultivar where relevant, unique accession number if available, and source (including location for collected wild specimens).	Not Applicable	
**Microbes:** provide species and strain, unique accession number if available, and source.	Not Applicable	

Human research participants	Information included in the manuscript?	In which section is the information available? (Reagents and Tools Table, Materials and Methods, Figures, Data Availability Section)
If collected and within the bounds of privacy constraints report on age, sex and gender or ethnicity for all study participants.	Not Applicable	

Core facilities	Information included in the manuscript?	In which section is the information available? (Reagents and Tools Table, Materials and Methods, Figures, Data Availability Section)
If your work benefited from core facilities, was their service mentioned in the acknowledgments section?	Yes	Acknowledgements

Design

Study protocol	Information included in the manuscript?	In which section is the information available? (Reagents and Tools Table, Materials and Methods, Figures, Data Availability Section)
If study protocol has been **pre-registered, provide DOI in the manuscript**. For clinical trials, provide the trial registration number **OR** cite DOI.	Not Applicable	
Report the **clinical trial registration number** (at ClinicalTrials.gov or equivalent), where applicable.	Not Applicable	

Laboratory protocol	Information included in the manuscript?	In which section is the information available? (Reagents and Tools Table, Materials and Methods, Figures, Data Availability Section)
Provide DOI OR other citation details if **external detailed step-by-step protocols** are available.	Not Applicable	

Experimental study design and statistics	Information included in the manuscript?	In which section is the information available? (Reagents and Tools Table, Materials and Methods, Figures, Data Availability Section)
Include a statement about **sample size** estimate even if no statistical methods were used.	Yes	Figure legends
Were any steps taken to minimize the effects of subjective bias when allocating animals/samples to treatment (e.g. **randomization procedure**)? If yes, have they been described?	Not Applicable	
Include a statement about **blinding** even if no blinding was done.	Not Applicable	
Describe **inclusion/exclusion criteria** if samples or animals were excluded from the analysis. Were the criteria pre-established?<br><br>If sample or data points were omitted from analysis, report if this was due to attrition or intentional exclusion and provide justification.	Yes	Information describing how particles were selected at different points of the image processing workflow are included in Materials and Methods.
For every figure, are **statistical tests** justified as appropriate? Do the data meet the assumptions of the tests (e.g., normal distribution)? Describe any methods used to assess it. Is there an estimate of variation within each group of data? Is the variance similar between the groups that are being statistically compared?	Not Applicable	

Sample definition and in-laboratory replication	Information included in the manuscript?	In which section is the information available? (Reagents and Tools Table, Materials and Methods, Figures, Data Availability Section)
In the figure legends: state number of times the experiment was **replicated** in laboratory.	Yes	Figure legend
In the figure legends: define whether data describe **technical or biological replicates**.	Yes	Figure legend

Ethics

Ethics	Information included in the manuscript?	In which section is the information available? (Reagents and Tools Table, Materials and Methods, Figures, Data Availability Section)
Studies involving **human participants**: State details of **authority granting ethics approval** (IRB or equivalent committee(s), provide reference number for approval.	Not Applicable	
Studies involving **human participants**: Include a statement confirming that **informed consent** was obtained from all subjects and that the experiments conformed to the principles set out in the WMA Declaration of Helsinki and the Department of Health and Human Services Belmont Report.	Not Applicable	
Studies involving **human participants:** For publication of **patient photos**, include a statement confirming that consent to publish was obtained.	Not Applicable	
Studies involving experimental **animals**: State details of **authority granting ethics approval** (IRB or equivalent committee(s), provide reference number for approval. Include a statement of compliance with ethical regulations.	Yes	Matherials and Methods
Studies involving **specimen and field samples:** State if relevant **permits** obtained, provide details of authority approving study; if none were required, explain why.	Not Applicable	

Dual Use Research of Concern (DURC)	Information included in the manuscript?	In which section is the information available? (Reagents and Tools Table, Materials and Methods, Figures, Data Availability Section)
Could your study fall under dual use research restrictions? Please check biosecurity documents and list of **select agents and toxins** (CDC): https://www.selectagents.gov/sat/list.htm	Not Applicable	
If you used a select agent, is the security level of the lab appropriate and reported in the manuscript?	Not Applicable	
If a study is subject to dual use research of concern regulations, is the name of the **authority granting approval and reference number** for the regulatory approval provided in the manuscript?	Not Applicable	

Reporting

The MDAR framework recommends adoption of discipline-specific guidelines, established and endorsed through community initiatives. Journals have their own policy about requiring specific guidelines and recommendations to complement MDAR.

Adherence to community standards	Information included in the manuscript?	In which section is the information available? (Reagents and Tools Table, Materials and Methods, Figures, Data Availability Section)
State if relevant guidelines or checklists (e.g., **ICMJE, MIBBI, ARRIVE, PRISMA**) have been followed or provided.	Not Applicable	
For **tumor marker prognostic studies,** we recommend that you follow the **REMARK** reporting guidelines (see link list at top right). See author guidelines, under 'Reporting Guidelines'. Please confirm you have followed these guidelines.	Not Applicable	
For **phase II and III randomized controlled trials**, please refer to the **CONSORT** flow diagram (see link list at top right) and submit the CONSORT checklist (see link list at top right) with your submission. See author guidelines, under 'Reporting Guidelines'. Please confirm you have submitted this list.	Not Applicable	

Data Availability

Data availability	Information included in the manuscript?	In which section is the information available? (Reagents and Tools Table, Materials and Methods, Figures, Data Availability Section)
Have **primary datasets** been deposited according to the journal's guidelines (see 'Data Deposition' section) and the respective accession numbers provided in the Data Availability Section?	Yes	Data Availability Section.
Were **human clinical and genomic datasets** deposited in a public access-controlled repository in accordance to ethical obligations to the patients and to the applicable consent agreement?	Not Applicable	
Are **computational models** that are central and integral to a study available without restrictions in a machine-readable form? Were the relevant accession numbers or links provided?	Not Applicable	
If publicly available data were reused, provide the respective **data citations in the reference list**.	Yes	Reference list in the main text
